# Ecotypic differentiation under farmers' selection: Molecular insights into the domestication of *Pachyrhizus* Rich. ex DC. (Fabaceae) in the Peruvian Andes

**DOI:** 10.1111/eva.12472

**Published:** 2017-03-23

**Authors:** Marc Delêtre, Beatriz Soengas, Prem Jai Vidaurre, Rosa Isela Meneses, Octavio Delgado Vásquez, Isabel Oré Balbín, Monica Santayana, Bettina Heider, Marten Sørensen

**Affiliations:** ^1^ Département Hommes, Natures, SociétésMuséum National d'Histoire NaturelleParisFrance; ^2^ Instituto de EcologiaUniversidad Mayor de San AndrésHerbario Nacional de BoliviaLa PazBolivia; ^3^ Museo Nacional de Historia NaturalUnidad de BotánicaLa PazBolivia; ^4^ Facultad de AgronomíaUniversidad de la Amazonía PeruanaIquitosPerú; ^5^ Programa de Investigaciones para el Aprovechamiento Sostenible de la BiodiversidadInstituto de Investigaciones de la Amazonía PeruanaIquitosPerú; ^6^ Genetics, Genomics and Crop ImprovementInternational Potato Center (CIP)LimaPerú; ^7^ Department of Plant and Environmental SciencesKobenhavns UniversitetFrederiksbergDenmark

**Keywords:** approximate Bayesian computation, ecotypic differentiation, landscape genetics, *Pachyrhizus*, plant domestication, progenitor–derivative speciation, yam beans

## Abstract

Understanding the distribution of crop genetic diversity in relation to environmental factors can give insights into the eco‐evolutionary processes involved in plant domestication. Yam beans (*Pachyrhizus* Rich. ex DC.) are leguminous crops native to South and Central America that are grown for their tuberous roots but are seed‐propagated. Using a landscape genetic approach, we examined correlations between environmental factors and phylogeographic patterns of genetic diversity in *Pachyrhizus* landrace populations. Molecular analyses based on chloroplast DNA sequencing and a new set of nuclear microsatellite markers revealed two distinct lineages, with strong genetic differentiation between Andean landraces (lineage A) and Amazonian landraces (lineage B). The comparison of different evolutionary scenarios for the diversification history of yam beans in the Andes using approximate Bayesian computation suggests that *Pachyrhizus ahipa* and *Pachyrhizus tuberosus* share a progenitor‐derivative relationship, with environmental factors playing an important role in driving selection for divergent ecotypes. The new molecular data call for a revision of the taxonomy of *Pachyrhizus* but are congruent with paleoclimatic and archeological evidence, and suggest that selection for determinate growth was part of ecophysiological adaptations associated with the diversification of the *P. tuberosus*–*P. ahipa* complex during the Mid‐Holocene.

## 1 Introduction

Evolution under continuous human selection has caused domesticated plants to evolve new traits and become adapted to novel environmental conditions often strikingly different from those of their wild progenitors. In legume crops, the change from indeterminate to determinate growth habit between wild and cultivated forms is one of the traits frequently associated with this “domestication syndrome” (Koinange, Singh, & Gepts, 1996). This is, in part, because plant architectural traits influence the partitioning of plant resources between vegetative and reproductive growth (Huyghe, 1998). By modifying growth habit, farmers can induce the plant to increase whole‐plant resource allocation to vegetative or reproductive parts, and select for different ecotypes adapted to contrasted environmental conditions. Determinacy is a particularly desirable trait because a compact growth habit is associated with shorter growth cycle, which is advantageous in short‐season environments (Kwak, Toro, Debouck, & Gepts, 2012). Indeterminate growth, in contrast, induces a prolonged competition for whole‐plant resources between reproductive and vegetative parts, but is better adapted to tropical environments, where constant humidity allows continuous plant growth and confers a competitive advantage as plants must compete for light.

Yam beans (*Pachyrhizus* Rich. ex DC., Fabaceae) are a good model for studying the need for manipulating whole‐plant resource allocation and its role in driving the evolution of plant architectural traits during plant domestication. Unlike most legume crops, yam beans are grown not for their seeds, which contain high levels of toxic polyphenols, but for their tuberous roots, which have high starch, vitamin, and protein contents as well as high levels of micro‐ and macronutrients (Ramos‐de‐la‐Peña, Renard, Wicker, & Contreras‐Esquivel, 2013).

For many plants, developing tuberous roots is a common strategy to survive protracted drought, and Harris (1972) suggested that most tropical root crops were domesticated in regions with marked seasonality before they were introduced in the more humid tropics. In root and tuber crops, most of which are propagated vegetatively, roots usually monopolize most plant resources (Kooman & Rabbinge, 1996). In legume plants, however, whole‐plant resource allocation is determined mostly by the sink effect exerted by the developing seeds, the strength of which is proportional to the number of maturing pods on the plant, thereby influencing resources available for the rest of the plant (Bennet, Roberts, & Wagstaff, 2011). Because yam beans are grown only from seeds, farmers must find a compromise between producing seeds (the part used for propagation) and producing storage roots (the part used for consumption) (Høgh‐Jensen, Mora, Morera, & Sørensen, 2008; Nielsen & Sørensen, 1998). The evolutionary importance of this tradeoff for the domestication and diversification of yam beans, however, has not been documented.

Yam beans have received increasing attention in recent years for the high‐quality starch and high nutritional value of their roots (Doporto, Mugridge, García, & Viña, 2011; López et al., 2010). However, their potential for crop improvement is limited by a lack of data on genetic resources in the genus (Santayana et al., 2014) and by confusion around phylogenetic relationships between *Pachyrhizus* species that are still not fully resolved.

The genus *Pachyrhizus* comprises five species (Sørensen, 1988), three of which are native to South America: (i) *P. panamensis* R.T. Clausen, which is only known in the wild; (ii) *P. tuberosus* (Lam.) Spreng., which is known both in the wild and as a cultivated species; and (iii) *P. ahipa* (Wedd.) Parodi, which is endemic to Bolivia and known exclusively as a cultivated species. The most widespread species, *P. tuberosus*, forms a complex of four cultivar groups: (i) “Ashipa,” (ii) “Chuin,” (iii) “Yushpe,” and (iv) “Jíquima.” Cultivars differ in leaf morphology, ecology, and traditional cultivation methods (Sørensen, Døygaard, Estrella, Kvist, & Nielsen, 1997). The most common cultivar group, Ashipas, are found in Ecuador, Peru, Bolivia, and Brazil. In Ecuador, Ashipas are known as “Iwa,” “Capamu,” or “Namau.” In Brazil, where their cultivation is now marginal, Ashipas are known as “Jacatupé” or “Feijão‐macuco” (Da Silva, Da Silva Filho, & Ticona‐Benavente, 2016). Ashipas are usually grown in slash‐and‐burn agroecosystems, in parts of the forest that are never inundated (*terra firme*). In contrast, Chuins and Yushpe thrive on rich alluvial soils and are grown on floodplains (*várzea*) that are inundated for 4–6 months a year (Oré Balbín, Sørensen, Kvist, & Delgado Vasquez, 2007). Chuins are found along the Marañón and Ucayali rivers in Peru, while Yushpes are cultivated only along the Ucayali River (Oré Balbín et al., 2007). The last cultivar group, Jíquima, is endemic to seasonally dry tropical forests (SDTFs) of the Guayas and Manabí provinces, in coastal Ecuador. Unlike other *P. tuberosus* cultivars, all of which are indeterminate, the Jíquima cultivar is a bushy plant with determinate growth, very similar to the Andean yam bean, *P. ahipa*, another endemic to SDTFs, which is found only in the semiarid inter‐Andean valleys of Bolivia and northern Argentina.

Seasonally dry tropical forests are characterized by <1,600 mm of annual rainfall and a marked dry season of 5 months at least, when total rainfall drops below 100 mm (Gentry, 1995). Environmental conditions in these regions are particularly suited for the evolution of tuberous roots in plants (Harris, 1972), and SDTFs have been proposed as important centers of origin for Andean roots and tuber crops (Debouck et al., 1995; Pearsall, 2008).

Little is known about the origins and history of South American yam beans. The origin of the Bolivian “Ajipa” (*P. ahipa*), in particular, is unknown, as no wild parent has yet been identified. This study represents the most comprehensive molecular study to date on the genus *Pachyrhizus* and aims at shedding light on the domestication history of yam beans in South America. We present new molecular evidence based on chloroplast DNA sequencing and a set of new nuclear microsatellite markers. Using a landscape genetic approach and approximate Bayesian computation (ABC), we examine correlations between environmental factors and phylogeographic patterns of genetic diversity within *Pachyrhizus* landrace populations, and evaluate different scenarios for the diversification of yam beans in the Peruvian Andes. Results are discussed through the lens of historical ecology.

## 2 Material and methods

### 2.1 Sampling, genotyping, and DNA sequencing

We analyzed 244 accessions (wild and cultivated) from field and herbarium specimens spanning the whole South American distribution range of the genus (Table S1). Total genomic DNA was extracted from 20 mg of lyophilized leaf tissue using NucleoSpin 96 Plant kits (Macherey‐Nagel, Hoerdt, France), following the manufacturer's instructions. Purified DNA was eluted in a final volume of 200 μl, and final concentration was checked using a NanoDrop ND‐1000 spectrophotometer (Labtech, Palaiseau, France). Genetic diversity was analyzed using the 17 nuclear Simple Sequence Repeats (SSRs) designed by Delêtre, Soengas, Utge, Lambourdière, and Sørensen (2013). PCR amplification, multiplexing, and genotyping were carried out following Delêtre et al. (2013). Three loci (AIP05, AIP09, and AIP34) showed no polymorphism across all species and were discarded.

Two fast evolving chloroplast DNA regions, 3′trnH‐5′psbA (Shaw et al., 2005) and 3′trnQ^UUG^‐5′rps16 (Shaw, Lickey, Schilling, & Small, 2007), were also tested for inter‐ and intraspecific polymorphism on a subset of 58 accessions spanning the entire distribution range of South American *Pachyrhizus* species. PCR was performed in 20 μl final volume, each containing 1 μl (~100 ng) of DNA template, 1× CoralLoad PCR Buffer, 1.5 mM MgCl_2_, 5% DMSO, 0.1 μg/μl BSA, 0.16 μM each of both forward and reverse primers (Eurofins MWG Operon, Ebersberg, Germany), dNTPs at 66 μM final concentration each, and 0.5 units of Taq DNA Polymerase (Qiagen, Hilden, Germany). For both regions, PCR cycling conditions were 7 min at 94°C, followed by 35 cycles of 1 min at 94°C, 1 min at 55°C, and 2 min at 72°C. Amplification ended with 10 min at 72°C. PCR products were purified and sequenced in both directions at the Eurofins sequencing facility. Sequences were aligned and manually edited using CodonCode Aligner 6.0.2 (CodonCode Corporation, Dedham, MA, USA). Sequences were deposited in GenBank under the accession numbers KT923495–KT923560 and KT924295–KT924350 (Table S1).

### 2.2 Phylogeographic patterns of genetic diversity

We used BAYESCAN 2.5 (Foll & Gaggiotti, 2008) to carry out neutrality tests for microsatellite markers and detect outliers signaling loci under selection. BAYESCAN assumes a model in which a common migrant pool exchanges genes with a number of subpopulations that differ from the common gene pool according to a population‐specific *F*_ST_. The difference in allele frequency between this common gene pool and each subpopulation is measured by a subpopulation‐specific *F*_ST_ coefficient. Although yam beans are predominantly self‐pollinating (Sørensen, 1996), which can increase the rate of false positives, BAYESCAN was shown to perform well with selfing populations when many (small) populations are considered (De Mita et al., 2013). Sixteen subpopulations were considered (Table S1). Default settings were used, and loci were considered outliers when their *q*‐value was <0.01 after applying a 1% false discovery rate correction. Loci under selection were removed from analysis.

Analyses of genetic variability were carried out using an extended dataset consisting of one copy of each unique multilocus genotype (MLG) identified at each sampling site (*n *=* *86) (Table S1). Mean number of alleles per locus (*A*), allelic richness corrected for sample size (*A*_R_), unbiased expected (*H*_E_) heterozygosity (Nei, 1978), and *F*‐statistics (Weir & Cockerham, 1984) were computed using FSTAT 2.9.3.2 (Goudet, 1995). Because *Pachyrhizus* populations were fixed for one allele at most loci, Jost's measure of estimated differentiation (*D*_est_), which estimates genetic differentiation based on shared alleles rather than based on mean heterozygosity (Jost, 2008), was used to carry out population differentiation tests with the R package DEMEtics (Gerlach, Jueterbock, Kraemer, Deppermann, & Harmand, 2010).

The structure of multilocus genetic diversity was explored using the Bayesian clustering method implemented in STRUCTURE 2.3.4 (Pritchard, Stephens, & Donnelly, 2000). STRUCTURE uses Markov chain Monte Carlo algorithms to group individuals in *K* clusters, assuming Hardy–Weinberg equilibrium and linkage equilibrium within each cluster, to reconstruct hypothetical ancestral populations. A narrow dataset consisting of one copy of each unique MLG (*n *=* *39) was used to avoid autocorrelation due to repeated MLGs. *K* was set to vary between 1 and 10. For each value of *K*, ten independent runs based on 100,000 Markov chain Monte Carlo iterations with a burn‐in period length of 10,000 generations were performed, assuming admixture and uncorrelated allele frequencies. No group priors were defined. The optimal number of clusters was determined using the Δ*K* method proposed by Evanno, Regnaut, and Goudet (2005). CLUMPP 1.1.2 (Jakobsson & Rosenberg, 2007) and DISTRUCT 1.1 (Rosenberg, 2004) were used to produce the final graphical outputs.

Discriminant analyses of principal components (DAPC) were performed in R (R Development Core Team, 2016) using the package Adegenet (Jombart, 2008). DAPC is a two‐step multivariate method which associates principal component analysis with discriminant analysis to achieve maximal discrimination of individuals into predefined groups (Jombart, Devillard, & Balloux, 2010). Unlike Bayesian approaches, which assumes that populations are at Hardy–Weinberg equilibrium and loci at linkage equilibrium, nonparametric methods make no assumptions and may be used to confirm results of Bayesian analyses when some conditions are not met (e.g., panmixia); DAPC is also better suited to unravel underlying genetic structuring in complex groups (Jombart et al., 2010). To infer the number of genetic clusters, DAPC uses sequential *K*‐means clustering of principal components. The “true” number of populations is determined in relation to a Bayesian information criterion (BIC). We retained all principal components (PCs) during the preliminary variable transformation to conserve all the variation in the original data. The number of PCs retained for the discriminant analysis was determined based on α‐scores (Jombart, 2008).

Phylogenetic relationships between *Pachyrhizus* populations were investigated by constructing a phylogenetic network based on Nei's minimum genetic distance (*D*_A_) (Nei, Tajima, & Tateno, 1983). POPULATIONS 1.2.32 (Langella, 1999) was used to produce the matrix of genetic distances and SPLITSTREE4 (Huson & Bryant, 2006) was used to create the split graphs using the Neighbor–Net algorithm. Finally, a minimum spanning haplotype network was constructed from the concatenated sequence data of the two chloroplast DNA (cpDNA) regions using HAPSTAR 0.7 (Teacher & Griffiths, 2011). Summary statistics were calculated using DnaSP 5.10 (Librado & Rozas, 2009).

### 2.3 Landscape genetics approach

GESTE 2.0 (Foll & Gaggiotti, 2006) was used to investigate the effect of environmental factors on the structure of genetic differentiation among *Pachyrhizus* landrace populations. GESTE uses a hierarchical Bayesian approach to estimate *F*_ST_ values for each local population and relate them to environmental factors using a generalized linear model. We first performed a PCA to identify a subset of key factors that captured most of the variance (Fig. S1). Based on this initial analysis, six environmental variables were retained: elevation (ALT), annual mean temperature (BIO1), isothermality (BIO3), temperature annual range (BIO7), annual precipitation (BIO12), and precipitation seasonality (BIO15). Longitude (LON) and latitude (LAT) were also included to test for isolation by distance. Additional occurrence data were compiled using herbarium specimens for which geographic coordinates were available, bringing the total to 169 unique presence points representing the 16 subpopulations (Table S1). Environmental data were extracted from the WorldClim database (Hijmans, Cameron, Parra, Jones, & Jarvis, 2005) at 30 arc‐second resolution using DIVA‐GIS 7.5.0 (Hijmans et al., 2012). Populations were represented by mean values of the eight bioclimatic variables. Burn‐in, thinning interval, sample size, and pilot runs were set to default values. The R package beanplot (Kampstra, 2008) was used to represent the observed distribution range of *Pachyrhizus* landrace populations along environmental gradients.

### 2.4 ABC analyses

We performed ABC analyses using the program DIYABC 2.1.0 (Cornuet et al., 2014) to compare different evolutionary scenarios for the origin of the Bolivian Ajipa (AC) and the Ecuadorian Jíquima (JI), and their relationship to wild (TW) and cultivated *P. tuberosus* (TC). Three sets of competing scenarios were compared (Fig. S2). In the first set, AC, JI, and TC diverged from distinct but closely related wild ancestors (partial genealogical link; scenarios S1–S5); in the second set, AC, JI, and TC were independently domesticated from the same wild ancestor (weak genealogical link; scenarios S6–S7); and in the third set, AC and JI diverged from TC (strong genealogical link; scenario S8). In all scenarios, dispersal events were assumed to be associated with strong genetic bottlenecks. Because herbarium specimens for wild *Pachyrhizus* were scarce, we introduced a fifth “ghost” population (Cornuet, Ravigne, & Estoup, 2010) to account for unsampled ancestral populations. All parameters were given wide uniform priors, except for bottleneck duration which was given a log‐uniform prior. Microsatellite loci were assumed to follow a symmetric generalized stepwise mutation model (Estoup, Jarne, & Cornuet, 2002), with a maximum range of 40 contiguous allelic states and a 10^−7^–10^−4^ mean mutation rate. As most of cpDNA variation consisted of indels, which the current version of DIYABC does not consider, cpDNA data showed too little variation to be informative and analyses were carried out with nuclear SSR data only. Details on model specifications, priors for demographic parameters, and locus‐specific mutation model parameters are given in Table S2.

For each scenario, 1 × 10^6^ datasets were simulated and eight pairwise summary statistics were computed (mean number of alleles across loci, mean gene diversity, mean allele size variance, pairwise *F*_ST_ and classification index, and δμ^2^). Confidence in scenario choice was assessed by evaluating type I and type II error rates over 100 pseudo‐observed datasets (PODs) simulated using parameter values drawn from prior distributions and LDA‐transformed summary statistics (Cornuet et al., 2010). Similarly, bias estimates were assessed by simulating 100 PODs under the best‐supported demographic scenario and using medians of demographic parameters drawn from the corresponding posterior distributions.

## 3 Results

### 3.1 Genetic variability among *Pachyrhizus* populations

Diversity indices for nuclear microsatellites and cpDNA are presented in Table 1. At the species level, genetic variability was highest among wild populations of *P. tuberosus*, which showed polymorphism at all loci but one (Fig. S3). In contrast, *P. ahipa* was fixed for one allele at 12 of the 14 loci analyzed.

**Table 1:** Comparison of nuclear and chloroplast diversity among *Pachyrhizus* populations

Region/Species	Status	Population	*N*	*G*	*A*_R_	*A*_P_	*H*_E_	*f*	*N*_CP_	*H*	*h*	*S*
Bolivia												
*P. ahipa*	Cult.	Aj‐N	14 (10)	4 (3)	1.027	2	0.026	0.846	6	1 (0)	—	—
		Aj‐S	5 (3)	2 (1)	1.014	1	0.013	1.000	5	1 (0)	—	—
*P. tuberosus*	Cult.	Yu‐L	3 (3)	2 (2)	1.114	—	0.095	1.000	3	1 (0)	—	—
		Yu‐H	4 (3)	3 (3)	1.061	—	0.054	1.000	2	1 (0)	—	—
Peru												
*P. tuberosus*	Cult.	As‐L	4 (3)	4 (4)	1.204	—	0.179	1.000	1	1 (0)	—	—
		As‐U	5 (5)	3 (3)	1.076	—	0.069	1.000	1	1 (0)	—	—
		As‐Y	4 (4)	3 (2)	1.163		0.143	1.000	3	3 (0)	0.667	4
		Ch‐L	9 (9)	2 (0)	1.026	—	0.025	1.000	5	1 (0)	—	—
		Ch‐U	7 (6)	2 (0)	1.031	—	0.029	1.000	3	1 (0)	—	—
		Yp	2 (2)	2 (1)	1.048	—	0.036	1.000	2	2 (0)	1.000	1
Brazil												
*P. tuberosus*	Cult.	Ja	4 (4)	1 (0)	1.000	—	—	—	3	1 (0)	—	—
Ecuador												
	Cult.	Ji	7 (7)	1 (1)	1.000	2	—	—	7	1 (0)	—	—
		Iw	8 (5)	7 (7)	1.169	—	0.159	1.000	7	2 (0)	0.286	4
*P. tuberosus*	Wild	Tw‐N	4 (4)	4 (4)	1.582	7	0.509	0.725	4	1 (0)	—	—
		Tw‐S	4 (3)	4 (4)	1.485	5	0.424	0.631	4	2 (1)	0.667	4
*P. panamensis*	Wild	Pw	2 (2)	2 (2)	1.524	2	0.393	0.800	2	2 (2)	1.000	5

*N*, sample size for nuclear diversity analyses (one copy of each unique multilocus genotype [MLG] per locality sampled). The number of sampled localities is indicated in brackets; *G*, number of distinct MLGs and number of private genotypes (in brackets); *A*_R_, allelic richness (mean rarefied allelic richness per locus); *A*_P_, number of private alleles; *H*_E_, unbiased expected heterozygosity; *f*, estimator of *F*_IS_; *N*_CP_, sample size for chloroplast diversity analyses; *H*, number of haplotypes and number of private haplotypes (in brackets); *h*, haplotype diversity; *S*, number of polymorphic sites. Aj, Ajipa (north and south); Yu, Yungas (lowlands and highlands); As, Ashipa (Loreto, Ucayali, Yungas); Ch, Chuin (Loreto and Ucayali); Yp, Yushpe; Ja, Jacatupé; Ji, Jíquima; Iw, Iwa; Tw, wild (north and south).

Matrices of pairwise genetic differentiation tests are given in Table S4. The average fixation index for all loci was high (*f *=* *0.907), reflecting the fact that most landraces consisted of few geographically discrete genotypes that were fixed for one allele at most loci and differed from each other by one or two alleles only (Table 1). The Ashipa cultivar group (*P. tuberosus*) showed the most variability, with the highest levels of genetic diversity recorded in Ecuador and Peru. In Peru, Ashipas cultivated by Tupi‐speaking groups (Cocama) in Dept. Loreto were distinct from Ashipas grown by Pano‐speaking groups (Shipibo) in Dept. Ucayali (*D*_est_ = 0.081, *p *<* *.01). Similarly, we found two genotypes of Chuins, one of which was predominantly found in Cocama villages (MLG 36), and the other in Shipibo villages (MLG 37). The two genotypes differed by one locus only (*D*_est_ = 0.028, *p *<* *.01) and were both also detected among Ashipas.

In Bolivia, Ajipa (*P. ahipa*) showed similarly low levels of genetic variability. Northern twining and southern bushy morphotypes were associated with distinct genotypes but differed by only one locus (*D*_est_ = 0.064, *p *<* *.01). Whereas northern accessions were fixed for AIP30_91_, southern accessions were fixed for allele AIP30_84_ (Fig. S4). Two rare alleles were also found, AIP17_48_ in the north, and AIP30_85_ in the south. Neither of these was detected in Peru, although both were present among wild populations from Ecuador.

Genetic analyses confirmed that all specimens collected in the Bolivian Yungas belonged to the Ashipa cultivar group (*P. tuberosus*), albeit to a genotype different from those cultivated in the Peruvian and Bolivian Amazon. This “Yungas type” was characterized by the fixation of two alleles, AIP17_50_ and AIP31_71_, which were otherwise detected among only four Peruvian Ashipas (TC356, TC357, TC364, and TC534) and in the Brazilian “Jacatupé” (Fig. S4).

The Ecuadorian Jíquima (*P. tuberosus*) was found to be genetically uniform, with only one genotype detected across the cultivar's entire distribution area. Although sharing unusual morphological characters, notably a determinate growth habit, Jíquima and Ajipa were genetically distinct (*D*_est_ = 0.308, *p *<* *.01). They were also both characterized by unique alleles at locus AIP30 (AIP30_84_ and AIP30_91_ for Ajipa, AIP30_95_ for Jíquima) and a shared allele at locus AIP23 (AIP23_69_) that was otherwise detected among wild accessions only. Genetic outlier analyses strongly suggested that this locus may be under disruptive selection (log_10_(*q* value) = −2.3, *p *>* *.99; Fig. S5), and AIP23 was therefore excluded from subsequent analyses. Ajipa was also characterized by a private allele at AIP28 (AIP28_32_) and AIP31 (AIP31_66_), while Jíquima was fixed for a private allele at AIP36 (AIP36_70_).

### 3.2 Phylogeographic structure of nuclear and chloroplast genetic diversity

**Figure 1:**
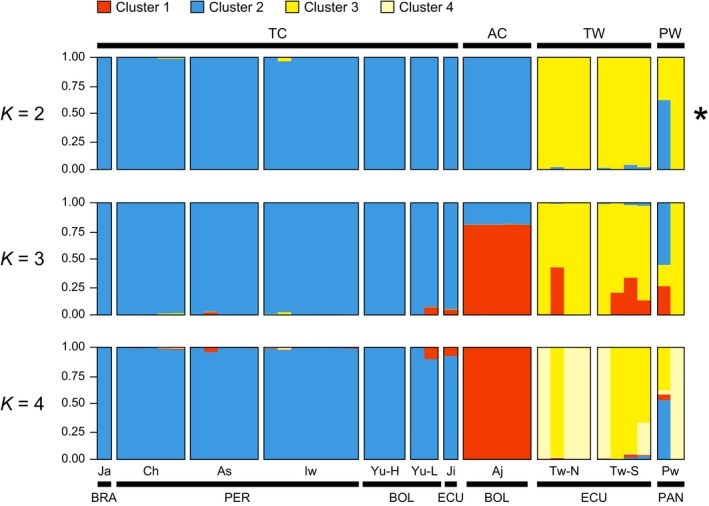
Unsupervised Bayesian clustering of individual *Pachyrhizus* multilocus genotypes (*n *=* *39), assuming increasing values from *K *=* *2 to *K *=* *4. The asterisk denotes the optimal number of clusters detected by STRUCTURE (Fig. S6). Individual plants are represented as thin vertical lines partitioned into colors corresponding to their membership to each of the *K* clusters. The upper horizontal black line represents species (AC,* P. ahipa*; TC, cultivated *P. tuberosus*; TW, wild *P. tuberosus*; PW,* P. panamensis*); the lower line represents the country of origin of accessions (BOL, Bolivia; BRA, Brazil; ECU, Ecuador; PAN, Panama; PER, Peru). Populations are identified as follows: Aj, Ajipa; Ji, Jíquima; Yu, Yungas (lowlands and highlands), As, Ashipa; Ch, Chuin; Ja, Jacatupé (Brazil); Iw, Iwa; Tw, wild *P. tuberosus* (Northern and Southern populations); Pw, *P. panamensis*. When including AIP23, Ajipa and Jíquima cluster together (data not shown)

Bayesian structuring analyses suggested that data were best represented by two populations. At *K *=* *2, wild *P. tuberosus* accessions (TW) clustered apart from cultivated accessions (TC and AC). At *K *=* *4, STRUCTURE separated *P. ahipa* (AC) from *P. tuberosus* (TC) and further distinguished two wild subpopulations (Figure 1). The only two accessions of *P. panamensis* (PW) available, PW055 (from Panama) and PW056 (from Ecuador), clustered both with TW, although PW056 showed signs of admixture with TC.

**Figure 2:**
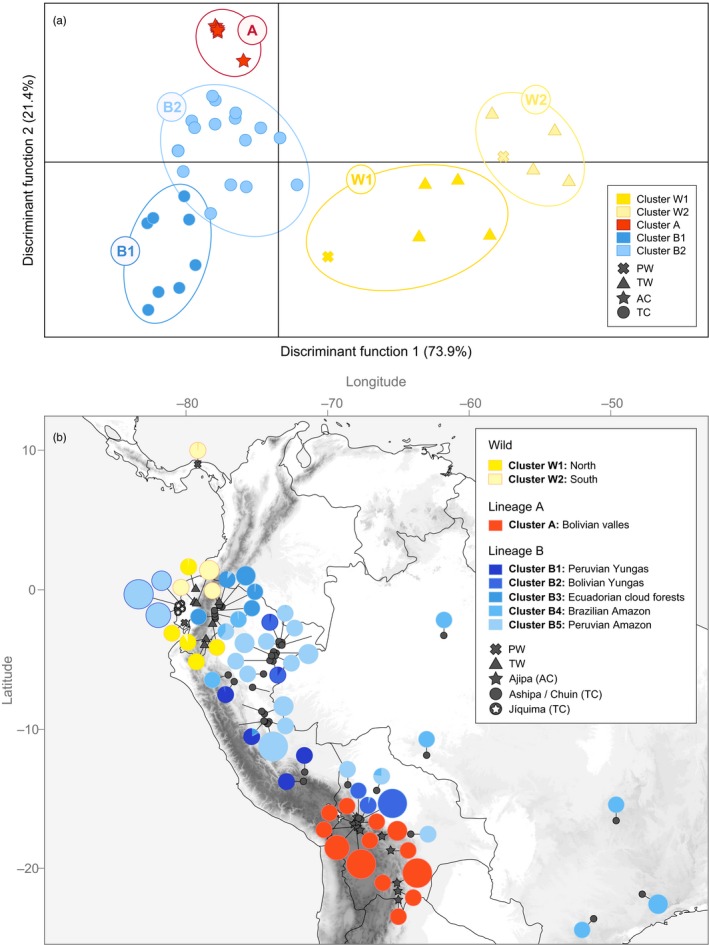
Discriminant analysis of principal components (DAPC) of *Pachyrhizus* accessions. (a) Scatterplot of unique multilocus genotypes (MLGs) based on DAPC clusters (*K *=* *5). Clusters are denoted by distinct colors, species by distinct symbols: *P. ahipa* (AC) is represented with stars, cultivated *P. tuberosus* (TC) with circles, wild *P. tuberosus* (TW) with triangles, and *P. panamensis* (PW) with crosses. (b) Geographic distribution and frequency of MLGs, showing inferred ancestry based on DAPC clusters. Circle size is proportional to the number of accessions

Results of DAPC analyses were congruent with the main clusters detected by STRUCTURE. Based on alpha‐scores, three PCs were retained for the discriminant analysis. The lowest BIC value was obtained for *K *=* *5 (Fig. S7). Most of the genetic structure was captured by the first two principal components (Figure 2a). The first axis, which explained 73.9% of the total variation, separated wild from cultivated accessions, while the second axis (21.4%) separated *P. ahipa* (cluster 1, hereafter referred as lineage A) from *P. tuberosus* (cluster 2, lineage B). As with STRUCTURE, DAPC separated wild *P. tuberosus* accessions (cluster 3, lineage W) into two distinct geographic subpopulations (W1 and W2), but additionally separated *P. tuberosus* cultivars in two subgroups (B1 and B2) (Figure 2a). To investigate finer levels of genetic structure, the DAPC was repeated for lineage B separately (Fig. S8). Five subpopulations were detected, each of which corresponding to a distinct subregion (Figure 2b).

**Figure 3:**
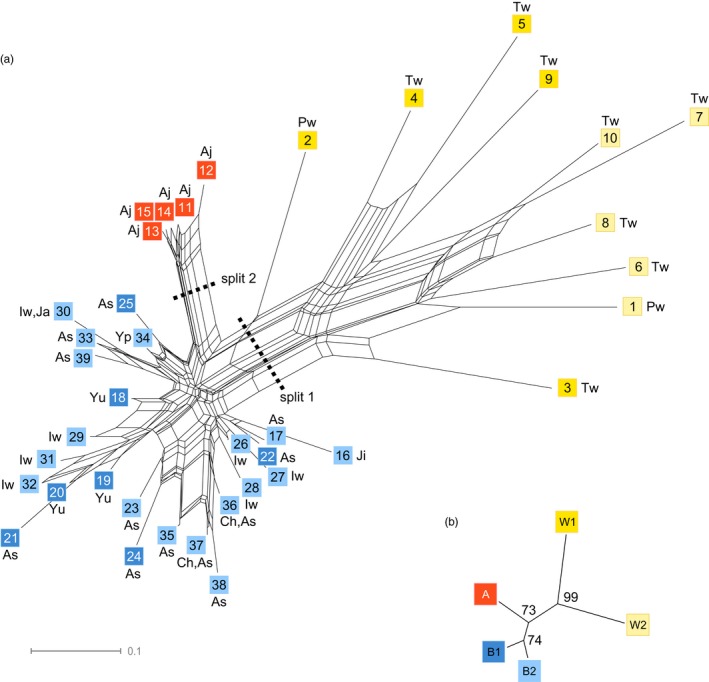
Unrooted phylogenetic network based on Nei's minimum genetic distance *D*_A_ (Nei et al., 1983). (a) Split graph of individual *Pachyrhizus* multilocus genotypes (MLGs). Splits 1 and 2 indicate strongly supported clusters. The scale bar indicates genetic distance. Nodes are colored according to the main genetic clusters identified by STRUCTURE and DAPC (lineages W, A, and B). Each MLG is identified by a number (see Table S1). Varietal types are also indicated (As, Ashipa; Aj, Ajipa; Ch, Chuin; Iw, Iwa; Ja, Jacatupé; Ji, Jíquima; Yu, Yungas type; Yp, Yushpe). *P. panamensis* and wild *P. tuberosus* accessions are indicated as Pw and Tw, respectively. (b) Neighbor‐joining tree based on Nei's minimum genetic distance *D*_A_ and 1,000 bootstrap resampling

Phylogenetic relationships among genetic clusters were further explored using Neighbor–Net algorithm. The topology of the phylogenetic network was in agreement with patterns identified using Bayesian and nonparametric clustering methods, with a primary split separating wild accessions (lineage W) from cultivated accessions, and a second split separating lineage A from lineage B (Figure 3).

**Figure 4:**
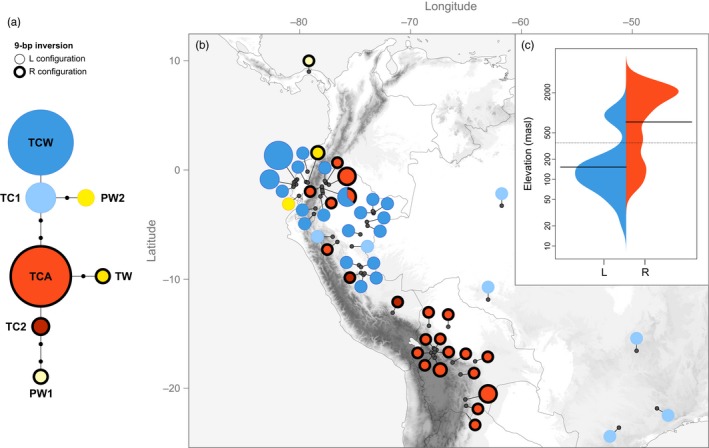
Patterns of chloroplast DNA variation in South American *Pachyrhizus* species. (a) Statistical parsimony network derived from the concatenated sequences of trnH‐psbA and trnQ‐rps16 (considering the 9‐bp inversion). Circle size is proportional to the frequency of the haplotype. The extent of evolutionary divergence between haplotypes is indicated by the number of black circles (mutation steps) on each line. (b) Geographic distribution of the seven combined cpDNA haplotypes. Pie charts show the frequency of haplotypes at each sampling site. Circle size is proportional to the number of accessions. Haplotypes carrying the R configuration are indicated with bold circles. (c) Beanplot of observed altitudinal distribution range for lineages L and R

Patterns of chloroplast DNA (cpDNA) diversity were generally concordant with nuclear data and confirmed the taxonomic status of *Pachyrhizus* species, except *P. ahipa* for which no specific haplotype was found. Seven chloroplast haplotypes were resolved from the concatenated sequences of trnH‐psbA and trnQ‐rps16 (Figure 4a). Eight variable sites (five substitutions, two indels, and one short inversion) were detected, only four of which were parsimony‐informative. Length variations at poly‐T microsatellite motifs were the only characters that distinguished the two haplotypes (TCW and TCA) resolved for the trnQ‐rps16 region. In trnH‐psbA, we detected a 9‐bp inversion region flanked on both sides by 16‐bp palindromic sequences (Fig. S9).

Homoplasious inversions of short sequences flanked by long inverted repeats are common at the intrageneric and intraspecific levels in trnH‐psbA, which can lead to overestimation of divergence among closely related species (Whitlock, Hale, & Groff, 2010). The distribution of the two configurations of the inversion among accessions was not random, however, and distinct geographic clusters could be identified (Figure 4b). While the L form characterized lowland *P. tuberosus* cultivars, the reverse complement (R) was predominantly found among highland Andean accessions (Figure 4c).

All nucleotide substitutions were linked to the presence/absence of this inversion. We detected two main haplotypes, TCW (carrying the L configuration) and TCA (carrying the R configuration). Most Peruvian accessions (TC) carried the TCW haplotype, while TCA was detected only in TC364. In contrast, all Bolivian accessions (AC and TC) carried the TCA haplotype. TCA also predominated among highland cultivars from Ecuador, while the lowland Jíquima group carried the TCW haplotype.

Although there was generally good agreement between the two chloroplast regions, with plants carrying either a combination of the haplotypes characteristic of *P. tuberosus* or of *P. ahipa*, Brazilian Jacatupé accessions carried the *P. tuberosus* haplotype for the trnH‐psbA region (L configuration), but the *P. ahipa* haplotype for the trnQ‐rps16 region, resulting in a distinct TC1 haplotype. TC1 was also detected in two Peruvian Ashipas collected near Tarapoto, TC359 and TC534. The only other accessions for which there was discordance between trnH‐psbA and trnQ‐rps16 were two Peruvian accessions, a Yushpe from Pucallpa (TC595) and an Ashipa collected near Cusco (TC537), both of which carried a unique combination of cpDNA haplotypes (TC2). Together with TC364, these were the only Peruvian accessions which carried the R form of the cpDNA inversion (Table S1).

### 3.3 Influence of environmental factors on the structure of genetic diversity

**Figure 5:**
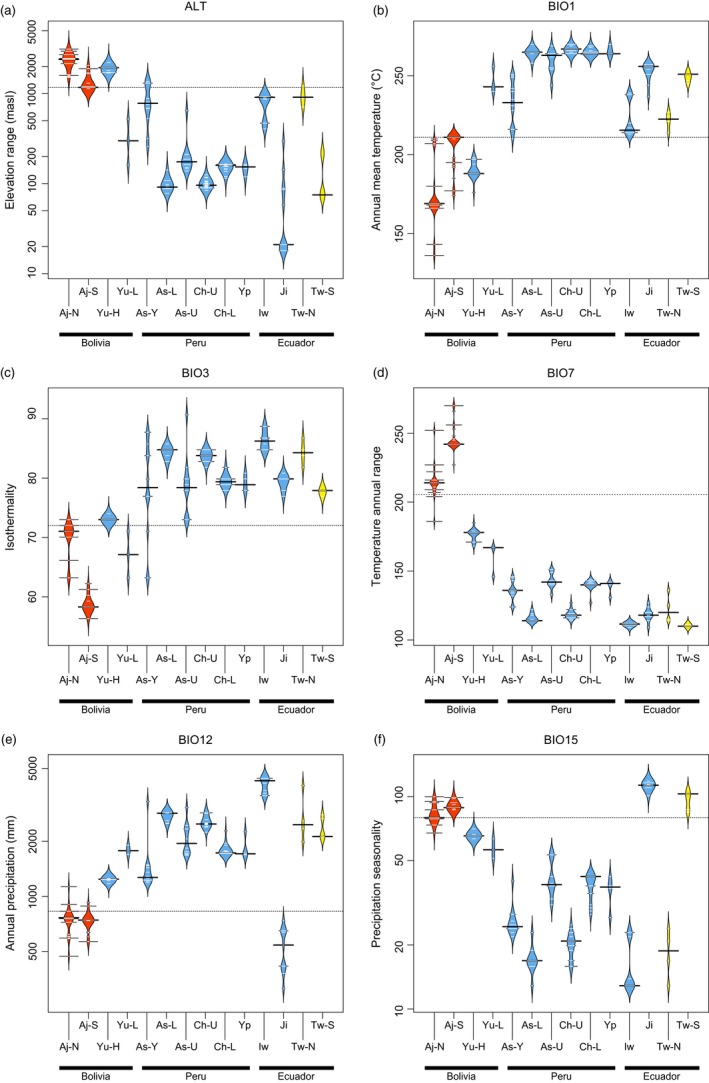
Comparison across *Pachyrhizus* landrace populations of their distribution range along the six environmental variables included in GESTE analyses: (a) elevation (ALT), (b) annual mean temperature (BIO1), (c) isothermality (BIO3), (d) temperature annual range (BIO7), (e) annual precipitation (BIO12) and (f) precipitation seasonality (BIO15). Fifteen landrace subpopulations were defined (Table S1) as follows: Aj, Ajipa (north and south); Yu, Yungas (lowlands and highlands); As, Ashipa (Loreto, Ucayali, Yungas); Ch, Chuin (Loreto and Ucayali); Yp, Yushpe; Ji, Jíquima; Iw, Iwa; Tw, wild (north and south). Brazilian accessions (Jacatupé), whose exact geographic origin is unknown, were not included. Wild populations are shown for comparison. Beanplots are colored according to the three main genetic clusters identified by STRUCTURE and DAPC (lineages A, B, and W). Individual observations are shown as small horizontal white lines within the estimated density trace. Dotted lines represent the median over all subpopulations

The relative importance of environmental factors on genetic differentiation between *Pachyrhizus* landrace populations was assessed using GESTE. Excluding wild populations, generalized linear regression models showed a positive correlation between precipitation seasonality (BIO15) and genetic differentiation between landrace populations, but no significant influence of elevation (ALT) or geographic distance (LON, LAT) on the structure of genetic diversity (Table 2). Precipitation seasonality had the highest cumulative posterior probability (*P *=* *.875), with a posterior probability of .129. Landrace populations segregated into “xeric” and “mesic” ecotypes, with a clear separation between populations belonging to either lineage A or lineage B for bioclimatic variables related to rainfall (Figure 5).

**Table 2:** (A) Posterior probabilities for the models including the eight bioclimatic variables (G1–G8) included in GESTE analyses. The best‐supported model is shown in bold. (B) Parameter estimates for the best‐supported model

Model	*P*	Factors included	*p*
(A)			
(null)		(constant)	.012
G1	.216	Annual mean temperature (BIO12)	.002
G2	.268	Isothermality (BIO3)	.005
G3	.276	Temperature annual range (BIO7)	.005
G4	.206	Annual mean precipitation (BIO12)	.017
**G5**	**.875**	**Precipitation seasonality (BIO15)**	**.129**
G6	.224	Elevation	.003
G7	.248	Longitude	.002
G8	.344	Latitude	.003

Parameter	Factor	Mean	Mode	95% HPDI
(B)				
α_0_	(constant)	1.34	1.35	[0.54, 2.10]
α_5_	Precipitation seasonality (BIO15)	1.15	1.08	[0.35, 2.01]
σ^2^		0.93	0.60	[0.16, 2.14]

*P*, sum of posterior probabilities for the models including the six bioclimatic variables; α, variable regression coefficient for each factor; 95% HPDI, 95% highest probability density interval; σ^2^, residual variance.

### 3.4 Divergence time between *P. ahipa* and *P. tuberosus*

**Figure 6:**
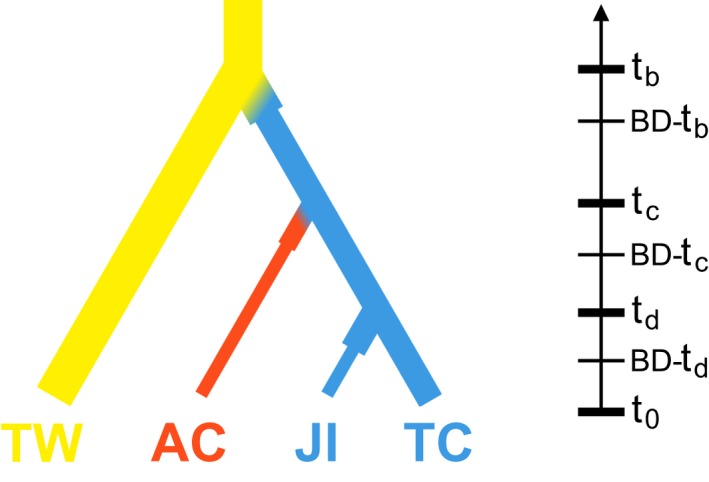
Best‐supported demographic scenario for the diversification of *Pachyrhizus* in South America, based on ABC analyses. Similar results were obtained when AIP23 was included (data not shown)

Using ABC, we analyzed phylogenetic relationships between *P. ahipa* and *P. tuberosus*. Analyses showed the strongest support for scenario S8 (posterior probability >99% (95% CI [0.988, 0.995]; Fig. S10), in which *P. ahipa* (AC) and Jíquima (JI) diverged from *P. tuberosus* (TC) (Fig. S2). Divergence time estimates suggest that TC diverged from TW ~7,600 years ago, while AC and JI diverged from TC ~3,900 and ~2,800 years ago, respectively (Figure 6 and Table 3). Confidence in scenario choice was strong (Table S5), with a type I error rate of 20%. Type II error rates were also very low (<5%), except in the case of S6 (10%). Bias and precision of parameter estimates are given in Table S6.

**Table 3:** Posterior estimates (mean and 95% confidence interval) of parameter values for the best‐supported evolutionary model based on ABC analyses (see Table S3 for details on priors and conditions). Divergence times are given in years, assuming a 1‐year generation time

Interpretation	Parameter	Mean (95% CI)
Effective population size		
TW	*N*_1_	15,400 [8,970, ,700]
TC	*N*_2_	1,570 [923, 1,980]
AC	*N*_3_	714 [175, 1,700]
JI	*N*_4_	456 [73, 1,590]
Divergence time		
TW→TC	*t*_*b*_	7,610 [3,890, 9,910]
TC→AC	*t*_*c*_	3,930 [1,220, 7,540]
TC→JI	*t*_*d*_	2,840 [770, 6,290]
Bottleneck duration		
TW→TC	BD*t*_*b*_	121 [1, 470]
TC→AC	BD*t*_*c*_	89.2 [1, 433]
TC→JI	BD*t*_*d*_	79.8 [1, 425]
Founding population size		
TC	*N*_*2*_ *F*	469 [23.2, 967]
AC	*N*_*3*_ *F*	301 [8.9, 879]
JI	*N*_*4*_ *F*	208 [5.4, 827]
Mutation rate	$\overset{¯}{\mu }$	3.94 × 10^−5^ [1.79 × 10^−5^, 7.76 × 10^−5^]
	$\overset{¯}{p}$	0.45 [0.13, 1.21]

## 4 Discussion

### 4.1 *Pachyrhizus ahipa* and *P. tuberosus* share a progenitor–derivative relationship

At present, *P. ahipa* is only known in cultivation in the subtropical east Andean valleys of Bolivia and northern Argentina (Ørting, Grüneberg, & Sørensen, 1996). Brücher (1989) suggested that *P. ahipa* might have been domesticated from wild material growing in the Bolivian Yungas, where the holotype specimen was collected (Mandon, 1856), but the species' taxonomic status remains unclear as no wild relative has been found. The striking genetic uniformity of *P. ahipa* throughout its entire distribution area, however, suggests ancient founder effects which, in turn, suggest that the crop may have been introduced into Bolivia only after it was domesticated.

Sørensen (1988) suggested that the distinct morphological characters of *P. ahipa* might be the result of farmers' selection and a long cultivation history. A possible scenario, supported by the ABC analyses (Figure 6), is that *P. ahipa* represents a case of “progenitor–derivative speciation” (PDS) (Crawford, 2010; Gottlieb, 2003). In this model of geographic speciation, a population “buds off” from its ancestral population and becomes adapted to a different habitat. Previous studies of progenitor–derivative (p–d) species pairs have shown that such species are generally genetically very similar, with the derivative species containing only a subset of the genetic diversity present in the progenitor. Derivative species are also characterized by (i) a narrow distribution range; (ii) unique morphological characters; and (iii) lack of variation in characters otherwise polymorphic in the putative ancestral population (Crawford, 2010).

Although PDS has mostly been applied to wild species, it can be extended to scenarios for the domestication and diversification of crop plants. PDS offers a plausible explanation for the drastic reduction in genetic diversity we found in *P. ahipa*, suggesting a strong genetic bottleneck following the plant's diffusion from its likely center of origin, in Ecuador, to the southeasternmost edge of the distribution range of the genus, in Bolivia. Morphological and molecular characters are congruent with a PDS scenario. *Pachyrhizus ahipa* shows several unique morphological characters that distinguish it from *P. tuberosus*, notably short, simple racemes with few flowers, which contrast with the complex racemes found in *P. tuberosus*; round legume pods; and the outward curling of the wing petals following anthesis, which is characteristic of the species (Sørensen, 1988). *Pachyrhizus ahipa* is also unique among yam beans in that it displays a sharp geographic contrast in growth habit, with twining types predominating in the north of Bolivia and bushy erect types prevailing in the south (Ørting et al., 1996). This wide range of infraspecific morphological and physiological plasticity (Sørensen, 1996) contrasts with a very low genetic variability across the species' distribution range. *Pachyrhizus ahipa* was fixed for one allele at most nuclear loci and shared its unique chloroplast DNA haplotype (TCA) with *P. tuberosus*, which further supports the hypothesis that *P. ahipa* and *P. tuberosus* share a progenitor–derivative relationship.

### 4.2 *Pachyrhizus ahipa* diverged from *P. tuberosus* through ecotypic differentiation

In most p–d species pairs, ecogeographic factors play a major role in preventing gene flow between the two species. Generalized linear regression models showed a clear segregation between *P. ahipa* (lineage A) and *P. tuberosus* (lineage B) and highlighted the importance of precipitation seasonality as the main factor affecting the structure of genetic diversity among *Pachyrhizus* landrace populations. Self‐pollination may have played an important role in facilitating the adaptation of *Pachyrhizus* cultivars to different environments by accelerating the fixation of beneficial alleles (Glémin & Ronfort, 2012; Ronfort & Glémin, 2013).

In northern Bolivia, *P. tuberosus* and *P. ahipa* are both locally known as “Ajipa,” but although they are found within a similar altitudinal range (1,500–2,000 m.a.s.l.), they never occur together in farmers' fields (Sørensen, 1996). Ashipas (*P. tuberosus*) are grown predominantly in the Yungas, a transitional zone between the Andean highlands and Amazon lowland rainforests characterized by warm tropical climate with no dry season, while Ajipa (*P. ahipa*) is only cultivated in the semiarid inter‐Andean valleys. Genetic analyses confirmed that all *P. tuberosus* accessions from the Yungas belonged to the Ashipa type. Like most Ashipas from Ecuador but unlike those from Peru, all Bolivian Ashipas carried the R form of the cpDNA inversion. Tapia and Sørensen (2003) showed that this “Yungas type” was also morphologically distinct from Peruvian Ashipas; while having the complex inflorescences typical of *P. tuberosus*, the growth habit, tuber shape and seed size, shape and color of Bolivian Ashipas are more similar to *P. ahipa* than they are to Peruvian Ashipas (Sørensen et al., 1997). Like other *P. tuberosus* cultivar groups, however, these Yungas accessions differ from *P. ahipa* by distinct flowering periods and a markedly longer vegetative growth (Sørensen et al., 1997; Tapia & Sørensen, 2003). These distinct phenological characteristics probably contributed to restrict gene flow between the two species. Hybridization experiments suggest a lack of absolute reproductive isolation between *P. ahipa* and *P. tuberosus* (Grüneberg, Freynhagen‐Leopold, & Delgado‐Váquez, 2003; B. Heider, E. Romero, R. Eyzaguirre, W. Grüneberg in preparation). This lack of reproductive barrier calls the taxonomical distinction between *P. ahipa* and *P. tuberosus* into question, but further supports the hypothesis that the two species share a progenitor–derivative relationship. Disjunct flowering periods and distinct flower morphology could be by‐products of selection by farmers for ecotypes adapted to different climatic conditions, that is, for shorter growth cycle, more advantageous in seasonal environments with a marked dry season. Such adaptive differentiation could have been facilitated by *Pachyrhizus* species being mostly self‐pollinating, thus accelerating the fixation of distinct alleles and unique morphological characters between the progenitor (*P. tuberosus*) and the derivate species (*P. ahipa*).

### 4.3 Convergent evolution of the Jíquima in Ecuador

Despite sharing many phenotypical characters with *P. ahipa*, the Ecuadorian Jíquima was genetically distinct and carried a different form of the 9‐pb cpDNA inversion. Ajipa and Jíquima shared only one allele at locus AIP23 and clustered together in STRUCTURE analyses when this locus was included, but no longer when the locus was removed. Neutrality tests showed AIP23 to be likely under positive selection, suggesting that morphological similarities between the two cultivars result from convergent evolution under farmers' selection for adaptations to similar environmental constraints rather than from a shared origin.

Approximate Bayesian computation analyses suggest that the Ecuadorian Jíquima diverged from TC about 2,800 years ago. Like *P. ahipa* in Bolivia, the current distribution range of the Jíquima cultivar is very limited and restricted to the Manabí and Guayas provinces of coastal Ecuador. The 460 mm annual precipitation rate in the region is lower than in any of the other habitats where *P. tuberosus* is found, but comparable to the 400–700 mm annual precipitation rate in Bolivian inter‐Andean valleys. While leaf shape differs between the two cultivars, Ajipa and Jíquima share important agromorphological characters, notably determinate growth and short racemes, but also photothermal neutrality (Sørensen, 1996), a trait associated with adaptation to higher elevations and/or drought‐prone environments (Kwak et al., 2012). Farming techniques are also similar between the two cultivars, which both require reproductive pruning. In areas where rainfall is strongly constrained by seasonality, a determinate growth habit is more advantageous to farmers as it shortens the growth cycle (Huyghe, 1998), but it compels farmers to prune flower buds to force plants to mobilize resources in increasing the size of tuberous roots. In contrast, constant humidity in tropical forests allows continuous plant growth and favors late flowering indeterminate genotypes, for which reproductive pruning is unnecessary.

### 4.4 Domestication and diversification of yam beans in South America

**Figure 7:**
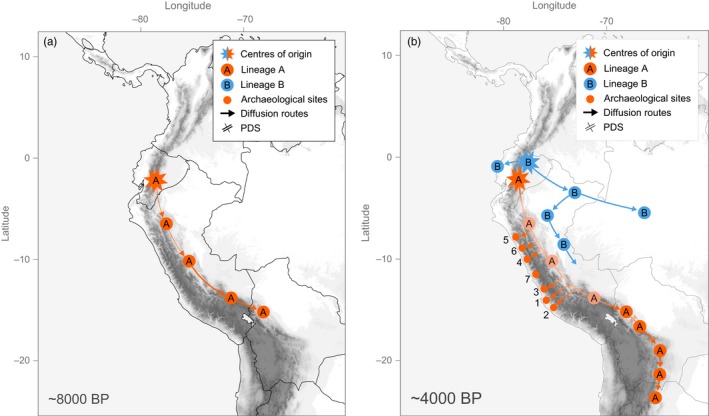
Proposed scenario for the domestication and diversification of the *Pachyrhizus tuberosus*–*Pachyrhizus ahipa* complex. Archeological sites with records of *Pachyrhizus* cultivation are indicated (1, Yacovleff, 1933; 2, Towle, 1952; 3, Engel, 1973 [4200–3500 BP], Jones, 1988 [~3300 BP]; 4, Grobman & Bonavia, 1978 [3800 BP]; 5, Ugent et al., 1982 [3800–3500 BP]; 6, Ugent et al., 1986 [AD 1500]; 7, Quilter et al., 1991 [3800–3500 BP]). (a) Domestication of *P. tuberosus* during the Early–Mid‐Holocene (~8000 BP) and diffusion of drought‐tolerant monotuberous ecotype (lineage A) with determinate growth across Peruvian and Bolivian Yungas. (b) Diffusion of mono‐/multituberous mesic cultivars with indeterminate growth (lineage B) in the Upper Amazon (~4000 BP) and diversification into Ashipa, Chuin, and Yushpe; few plants are also introduced into Brazil (Jacatupé); relocation of A ecotypes to refuge areas in Peruvian and Bolivian inter‐Andean valleys and differentiation through progenitor–derivative speciation under farmers' continuous selection

Based on the molecular evidence presented in this study, we propose a two‐phase scenario for the domestication and diversification of yam beans in the Peruvian Andes (Figure 7).

Approximate Bayesian computation analyses suggest that yam beans diverged from their wild relatives ~8,000 years ago, which coincides with the Early–Mid‐Holocene, a period of environmental transition and agricultural expansion in the Peruvian Andes (Pearsall, 2008; Piperno, 2006). Yam beans were probably domesticated in the dry forests of western Ecuador, where most wild specimens of *P. tuberosus* have been recorded (Sørensen et al., 1997), and where we also found the highest levels of genetic diversity.

There is strong evidence that reduced precipitations during the Mid‐Holocene led to significantly drier climatic conditions in the tropical Andes than at present, causing cloud forests to be replaced by semideciduous dry forests in ecotonal areas (Mayle & Power, 2008). This dry period would have been propitious to the domestication of drought‐tolerant ecotypes (lineage A, carrying the R form of the cpDNA inversion) and made possible their diffusion along the eastern slopes of the Andes (Figure 7a). Little is known about selective constraints on chloroplast intergenic spacers; the two chloroplast lineages we detected might represent distinct dispersal events or might be related to adaptations to different environments, with the R form becoming fixed preferentially in ecotypes selected for cultivation at higher elevations. The disjunct distribution of the L and R forms of the cpDNA inversion and vestigial traces of the R form in plants collected in the Peruvian Yungas clearly suggest however that the R form spread first along the eastern slopes of the Andes across Peru and into Bolivia, before it was replaced by the L form in the Peruvian Amazon.

Approximate Bayesian computation analyses suggest that *P. ahipa* diverged from *P. tuberosus* during the Late Holocene, ~4,000 years ago. This period corresponds to a transition toward wetter conditions in the Peruvian Amazon, and a reversion of dry forests to *várzea* rainforests (Mayle & Power, 2008). Better adapted to permanently humid climates, lowland cultivars (lineage B, carrying the L form) spread along the Peruvian Upper Amazon, replacing drought‐tolerant ecotypes whose cultivation shifted to refuge areas in Peruvian and Bolivian inter‐Andean valleys (Figure 7b).

Several landraces of Ashipas and Chuins have been described, the largest diversity of which is found in the Peruvian Amazon. “White Ashipas” have been recorded along the Huallaga, Marañón, and Ucayali rivers, while “yellow Ashipas” occur mainly along the Tigre River but are also found occasionally along the Nanay, Marañón, and Ucayali rivers. Similarly, farmers distinguish between “white,” “yellow,” and “purple” Chuins, but also between cultivars with high dry matter (DM) content, known as “Pitichuin,” and low DM varieties, known as “Cocotichuin.” Purple Pitichuins are the most common, while yellow and purple Cocotichuins are only known from Shipibo communities. Chuins are only found along the Ucayali River, where they are grown by Tupi‐speaking groups (Cocama) and Pano‐speaking groups (Shipibo), each of which grew distinct genotypes of Chuins. The diversity of unrelated vernacular names for Ashipa landraces in Peru and Ecuador suggests that Ashipas represent the ancestral landrace population from which all other cultivars derived (Sørensen et al., 1997). Molecular data seem to support this hypothesis, with Chuins and Jacatupé containing only a subset of the genetic variability found in Ashipas. In turn, the partitioning of morphological and genetic diversity into local landraces suggests serial founder effects accompanying the diffusion of lowland cultivars through the Peruvian Amazon.

Drought‐tolerant ecotypes were probably also brought down to the valleys on the western slopes on the Andes, where archeological evidence indicates that “Ajipa‐like” cultivars were cultivated on the Peruvian coast as early as 4000 BP (Figure 7b). Remains of tuberous roots found in mummy bundles at several archeological sites along the coast of Peru confirm that *Pachyrhizus* plants with determinate growth habit, very similar to *P. ahipa*, were cultivated by pre‐Columbian cultures in the Late Preceramic–Initial Period (3800–2900 BP) up until the Late Intermediate Period (AD 1000–AD 1500) (Towle, 1952; Ugent, Pozorski, & Pozorski, 1986; Yacovleff, 1933). Remains were dated to 3800–3500 BP (Engel, 1973; Grobman & Bonavia, 1978; Quilter et al., 1991; Ugent, Pozorski, & Pozorski, 1982) and AD 1500 (Ugent et al., 1986), but their taxonomic identity is still debated. Hawkes (1989) and Yacovleff (1933) identified the remains as Jíquima (*P. tuberosus*), whereas Ugent et al. (1982, 1986) identified the remains as Ajipa (*P. ahipa*).

The persisting ambiguity about the taxonomic status of these plant remains is mainly due to the absence of cultivars with determinate growth in Peru nowadays (Sørensen et al., 1997). Yam bean agriculture in Peru is strongly linked with the development of pre‐Columbian societies on the western Andean foothills during the Late Holocene. River catchment basins form oases of riparian dry forests along the Peruvian coast. Known as “lomas,” these forests depend on seasonal rainfall in the headwaters of Andean highlands. Lomas have played a major role in the history of pre‐Columbian cultures (Engel, 1973; Fehren‐Schmitz et al., 2014), and archeological records indicate they were focal points for the early onset of yam bean agriculture in the Peruvian Andes. However, these lowland populations probably disappeared as desertification progressed on the coast of Peru starting ~AD 500, forcing settlements to relocate into the middle and upper valleys of the Andean foothills (Fehren‐Schmitz et al., 2014; Schittek et al., 2015). Roots discovered in the Casma River valley and dated from ~AD 1500 (Ugent et al., 1986) indicate that *Pachyrhizus* cultivation was briefly resumed on the Peruvian coast during the Late Intermediate Period as climatic conditions improved and Chimú‐Inca resettled coastal lowlands for the last time (Fehren‐Schmitz et al., 2014; Mächtle & Eitel, 2013). This suggests that “Ajipa‐like” landraces were still cultivated in Peruvian intermontane valleys up until the 15th century, although molecular analyses would be needed to establish whether plant remains discovered in Peru are Ajipa or Jíquima roots, or—possibly—intermediate genotypes. The coastal province of Piura in northwestern Peru, where Emperaire and Friedberg (1990) reportedly collected one *P. ahipa* specimen (unconfirmed), and the Apurímac, Ene, and Mantaro river valleys in central eastern Peru, where Debouck et al. (1995) suggested that “ancestral” populations of *P. ahipa* might be found (also unconfirmed), should also be given priority for collecting germplasm which could, if it (still) exists, help resolve the history of *P. ahipa* in Peru.

Given the limited genetic variation in *P. tuberosus* and *P. ahipa* landrace populations, it is urgent to document and safeguard genetic resources in *Pachyrhizus* wild relatives. *Pachyrhizus panamensis*, in particular, represents an important taxonomic gap which needs to be addressed. Like Ajipa and Jíquima, *P. panamensis* distribution is restricted to SDTFs. Pollen grains of *P. ahipa* are morphologically distinct from pollen grains of *P. tuberosus* and support a close relationship between *P. ahipa* and *P. panamensis* (Sørensen, 1989), although this could also indicate an adaptation to similar environmental stress. Phylogenetic relationships between *P. panamensis, P. tuberosus,* and *P. ahipa* cannot be fully resolved, however, because of the rarity of *P. panamensis* herbarium specimens.

In conclusion, while gray areas remain, this study sheds a new light on phylogenetic relationships between *P. tuberosus* and *P. ahipa* and offers insights into the evolutionary history of yam beans in the Andes. Molecular data suggest the two species share a progenitor–derivative relationship, with environmental factors playing an important role in driving selection for divergent ecotypes adapted to contrasted environments. Future collection missions in Peru and Ecuador might bring new elements that will help refine the scenario we propose.

## Conflict of interest

The authors declare no conflict of interest.

## Data archiving statement

Data for this study are available from the Dryad Digital Repository: https://doi.org/10.5061/dryad.7gp80. Chloroplast DNA sequences were deposited in GenBank under accession numbers KT923495–KT923560 and KT924295–KT924350.
