# Supplementary material for: Ecotypic differentiation under farmers' selection: Molecular insights into the domestication of Pachyrhizus Rich. ex DC. (Fabaceae) in the Peruvian Andes
Source: Evol Appl. 2017 Mar 23;10(5):498–513. doi: 10.1111/eva.12472 (PMC5427675; doi:10.1111/eva.12472)
Supplement: Supplementary file 2 [file EVA-10-498-s002.docx]

Supporting Information

**Ecotypic differentiation under farmers’ selection: molecular insights in the domestication of *Pachyrhizus* DC. (Fabaceae) in the Peruvian Andes**

Marc Delêtre, Beatriz Soengas, Prem Jai Vidaurre, Rosa Isela Meneses, Octavio Delgado Vásquez, Isabel Oré Balbín, Monica Santayana, Bettina Heider, Marten Sørensen

**Metadata for Table S1** Geographic origin, genotypes, voucher specimen and accession numbers of *Pachyrhizus* samples used in analyses.

**Fig. S1** Principal component analysis (PCA) of the 19 bioclimatic variables used in landscape genetic analyses.

**Fig. S2** Graphical representation of the eight competing scenarios tested in ABC analysis.

**Table S2** Parameters for prior distribution and conditions used for ABC analyses.

**Table S3** Pairwise genetic differentiation tests.

**Fig. S3** Comparison across species of levels of genetic polymorphism (PIC).

**Fig. S4** Distribution of allelic diversity among *Pachyrhizus* landrace populations.

**Fig. S5** Results of the Bayesian outlier detection using BAYESCAN.

**Fig. S6** Optimal number of genetic groups detected by STRUCTURE.

**Fig. S7** Number of clusters detected by DAPC for the whole dataset.

**Fig. S8** Number of clusters detected by DAPC for lineage B.

**Fig. S9** Portion of trnH-psbA chloroplast intergenic spacer containing the 9-bp inversion.

**Figure S10** Comparison of estimated posterior likelihood for the eight competing scenarios.

**Table S4** Confidence in scenario choice.

**Table S5** Bias and precision of parameter estimates.

**Metadata for Table S1**

Herbaria from which accessions were obtained are identified according to the *Index Herbariorum* (Thiers 2011): CIP^1^ = International Potato Center; CP^2^ = Royal Veterinary and Agricultural University Herbarium, Copenhagen, Denmark; FHO = University of Oxford, Daubeny Herbarium, Oxford, United Kingdom; INPA = Instituto Nacional de Pesquisas da Amazônia, Manaus, Brazil; PAT = Laboratoire d'ethnobiologie-biogéographie, Paris, France; UAMH = Devonian Botanic Garden, University of Alberta, Canada; UEC = Universidade Estadual de Campinas, São Paulo, Brazil.

**Notes:**

^1^ Herbarium specimens collected as part of CIP Project Yam-bean, funded by the Belgian Development Cooperation (D43/JK).

^2^ Herbarium specimens collected as part of the ‘Yam Bean Project’ (1985-1999), funded under EU Science and Technology for Developing Countries program.


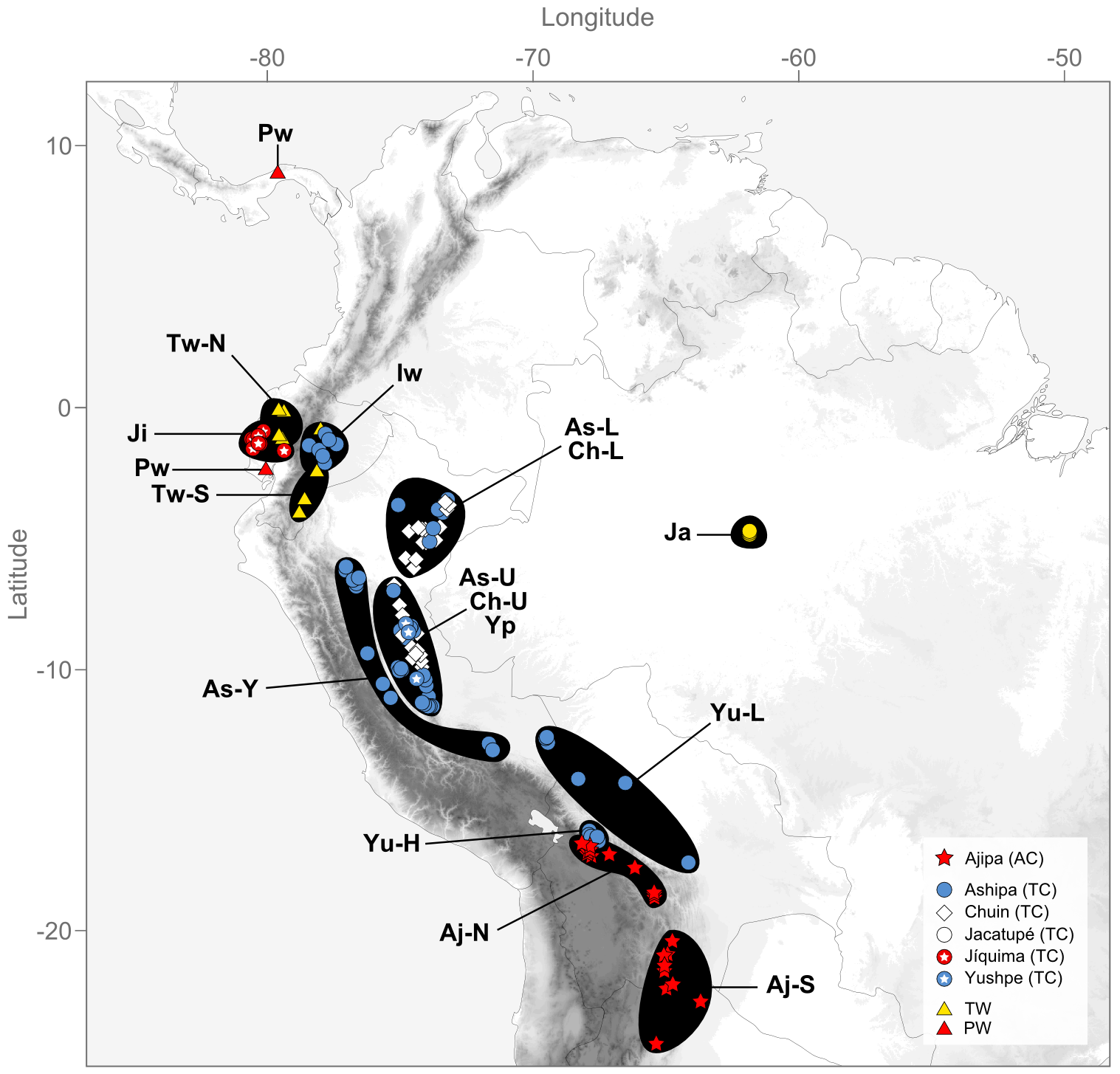


**Map.** Geographic distribution of the 169 unique points used in landscape genetic analyses. Based on ecogeographic, ethnographic, taxonomic and genetic data, 16 subpopulations were defined (see below). Species are identified as follows: AC, *P. ahipa*; TC, cultivated *P. tuberosus*; TW, wild *P. tuberosus*; PW, *P. panamensis*. Subpopulations are identified as follows: Aj, Ajipa (North and South); Yu, Yungas (Lowlands and Highlands); As, Ashipa (Loreto, Ucayali, Yungas); Ch, Chuin (Loreto and Ucayali); Yp, Yushpe; Ji, Jíquima; Iw, Iwa; Tw, wild (North and South); Ja, Jacatupé (details on geographic origin unavailable).

1. **Ecuador**

Subpopulation 1. Pw

*P. panamensis* is mostly found in deciduous forest in areas with at least one dry season per year lasting 2-3 months (250–1500 mm annual rainfall). The present-day distribution of the species is characterized by a large phylogeographic break, with populations known only from Central and Pacific Panama, and from the provinces of Guayas and El Oro in central Ecuador (Sørensen 1988). Only two accessions were available, and while these appeared to be genetically distinct, they were treated as a single population.

Subpopulation 2. Tw-N

Subpopulation 3. Tw-S

The natural habitat of *P. tuberosus* is tropical lowland or premontane rain forests with annual precipitation rates between 640 and 4100 mm. Wild populations of *P. tuberosus* are mostly found in Ecuador, within a wide altitudinal range (0-1900 masl) (Sørensen 1988). All wild specimens are indeterminate (Fig. S1). Molecular analyses showed genetic differences between accessions from the North (Tw-N) and the South (Tw-S), which were treated as distinct subpopulations.

*
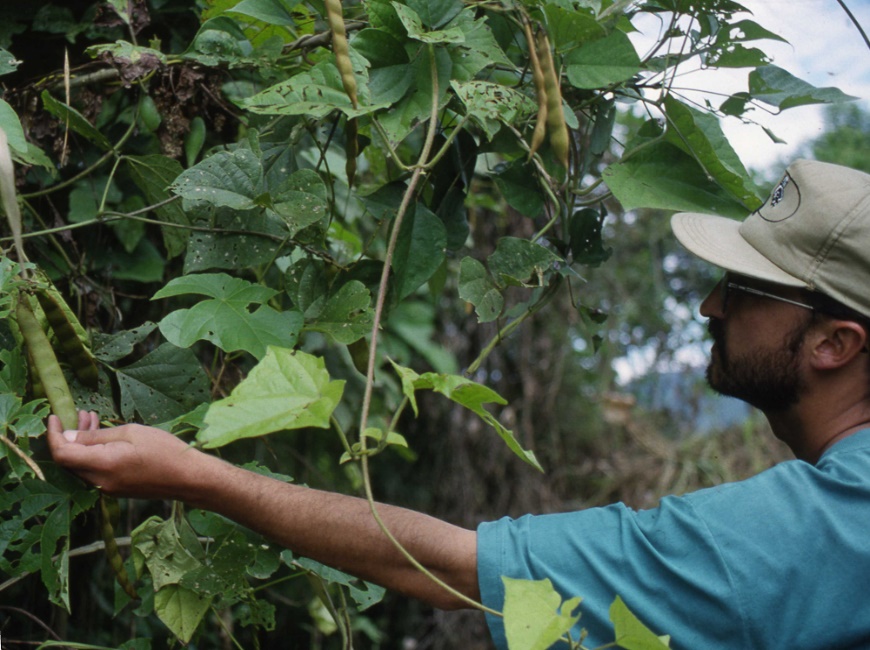
*
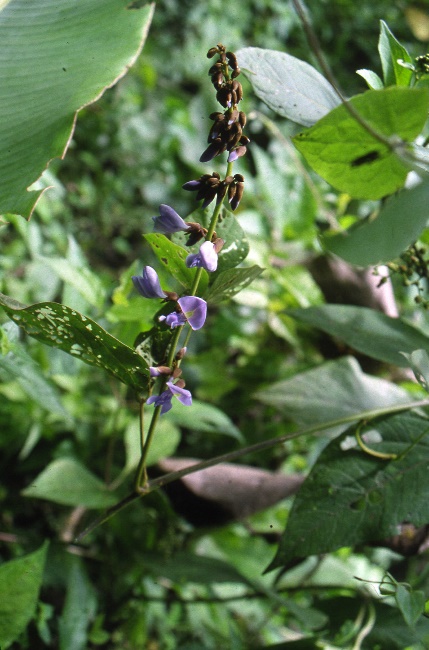


**B**

**A**

**Figure S1.** (A) Wild specimen of *P. tuberosus* from Prov. Morona Santiago, cantón Morona, Ecuador; (B) Inflorescences on a wild *P. tuberosus* plant (Photos: N, Mazon,1996).

Subpopulation 4. Iw

In Ecuador, *P. tuberosus* is cultivated mostly on the eastern slopes of the Andes. Sørensen et al. (1997) recorded several local landraces, as well as a rich diversity of vernacular names sharing no apparent linguistic relation (‘Iwa’, ‘Capamu’, ‘Namau’). This subregion was also characterized by higher allelic and cpDNA haplotypic diversity, and the presence in cultivated accessions of alleles otherwise found only in wild populations of *P. tuberosus*.

Subpopulation 5. Ji

Genetically and morphologically distinct from other *P. tuberosus* cultivars, Jíquima is only found in the Ecuadorian dry forests of the Manabí and Guayas provinces, in coastal Ecuador. Jíquima plants have determinate growth with short internodes and deeply lobed leaflets (Fig.S2A), as well as short, simple racemes with few flowers (Fig.S2B) that contrast with the indeterminate growth, complex racemes and entire leaflets observed in Amazonian cultivars (Tapia and Sørensen 2003).


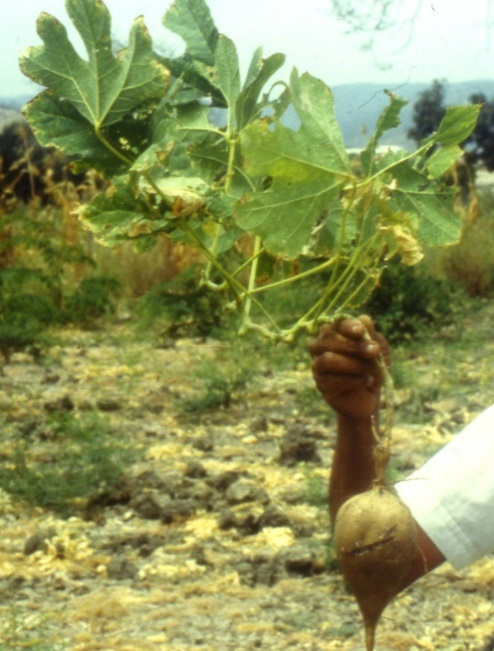

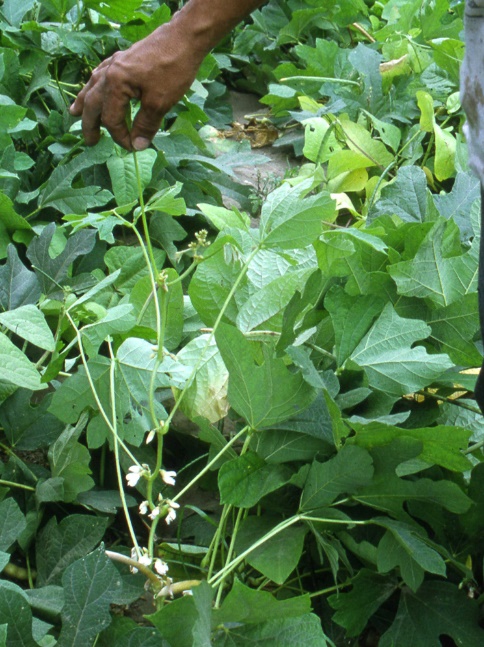


**B**

**A**

**Figure S2.** (A) Jíquima plant from Montecristi, Manabí province, Ecuador. The distinct determinate growth habit and deeply lobed leaves are clearly visible; (B) Simple inflorescence on a Jíquima plant (Photos: M. Sørensen and E. Heredia-Garcia, 1992).

1. **Peru**

Subpopulation 6. As-L

Subpopulation 7. As-U

Subpopulation 8. As-Y

In Peru, three cultivar groups of *P. tuberosus* have been described that differ in leaf morphology, agroecological requirements, traditional cultivation methods, and local uses: Ashipa, Chuin, and Yushpe (Sørensen et al. 1997). Some cultivars are further subdivided into distinct local landraces based on root colour and quality (Oré-Balbin et al. 2007). White Ashipas are found along the Huallaga, Marañon and Ucayali Rivers. Yellow Ashipas (Fig. S3) have also been described mainly along the Tigre River but can also be found occasionally along the Nanay, Marañon and Ucayali (Oré-Balbin et al. 2007). Molecular analyses showed that Ashipas cultivated by Tupi-speaking groups (Cocama) in Dept. Loreto (As-L) are distinct from Ashipas grown by Pano-speaking groups (Shipibo) in Dept. Ucayali (As-U). A third subpopulation, As-Y, encompasses all accessions from Peruvian Yungas.


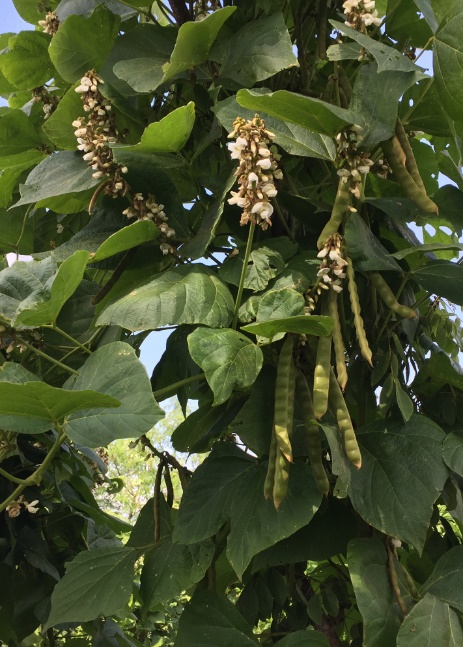

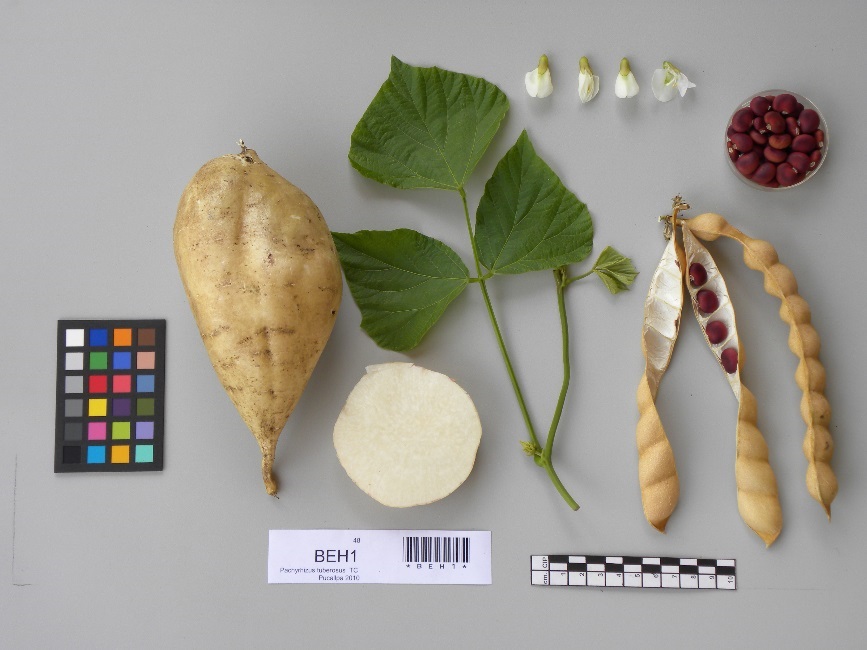


**B**

**A**

**Figure S3.** (A) (A) Ashipa amarilla from Dept. Loreto, Río Napo, Peru, showing a viny growth habit and complex inflorescences (Photo: S. Simonsen, 2016). (B) Ashipa root, leaves, flowers, seeds and legume pods (Photo: B. Heider, 2010).

| 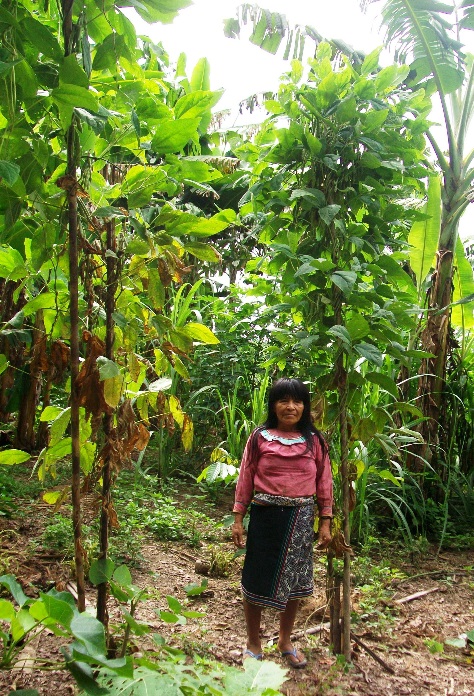  **A** | 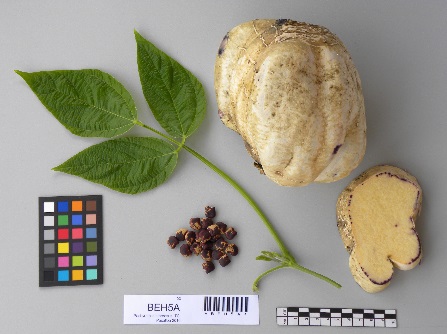  **B1**  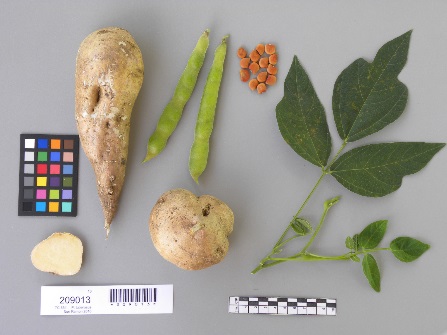  **B2** | 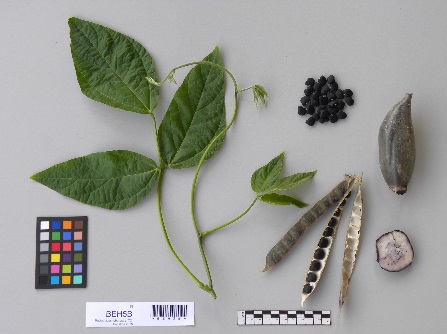  **C1**  **C1**  **B1**  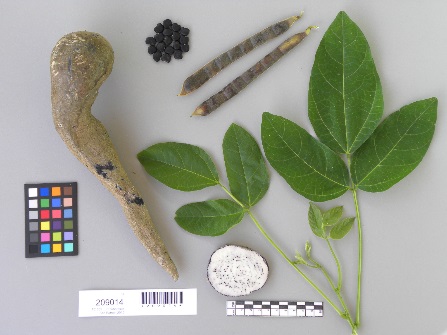  **C2** |
| --- | --- | --- |

**Figure S4.** (A) Chuin from Ucayali, Peru (Photo: B. Heider, 2010); (B1,B2) Yellow chuin (root, leaves, seeds and legume pods); (C1,C2) Purple chuin (Photo: B. Heider, 2010).

Subpopulation 9. Ch-L

Subpopulation 10. Ch-U

Unlike Ashipas, which are usually cultivated in slash-and-burn agricultural systems on so-called *‘terra firme’*, Chuins (Fig. S4) are grown on alluvial seasonally flooded fields (‘playas’) (Sørensen et al. 1997; Oré Balbín et al. 2007). Oré-Balbin et al. (2007) distinguished several landraces, categorized into two subgroups: Pitichuin (cultivars with high dry matter [DM] content) and Cocotichuin (low DM varieties). Within each subgroup, Amerindian farmers also distinguish among white, yellow, and purple Chuin, based on the colour of the root. Purple Pitichuin is the most widespread, whereas Yellow and purple Cocotichuin are only known from Shipibo communities. As with Ashipas, we found that Chuins grown in Cocama villages (Ch-L) were genetically distinct from Chuins grown in Shipibo villages (Ch-U). The town of Requena marks approximately the limit between the two distinct ethnolinguistic regions. We distinguished between the two subpopulations to reflect genetic and ethnolinguistic differences between accessions from Ucayali and Loreto.

Subpopulation 11. Yp

Yushpe is endemic to Peru and cultivated exclusively by Shipibo people along the Ucayali River. This elusive cultivar was first reported by Tessmann in 1928 and was re-discovered in 1999 (Oré Balbín et al. 2007). Yushpe is morphologically distinct from the other *P. tuberosus* cultivars, and although only two accessions were available, there were treated as a distinct subpopulation.

1. **Bolivia**

Subpopulation 12. Aj-N

Subpopulation 13. Aj-S

Known only from cultivation, *P. ahipa* is grown between 1500 and 2500m a.s.l. in the seasonally dry inter-Andean valleys of Bolivia and northern Argentina. Like Jíquima plants in Ecuador, Ajipa plants are determinate and have simple inflorescences with short racemes (Fig. S5). *Pachyrhizus ahipa* displays a sharp geographical contrast in growth habit, with twining types in the north of Bolivia (Aj-N) and bushy erect types in the south (Aj-S) (Ørting et al. 1996; Sørensen et al. 1997). Genetic analyses showed that the two morphotypes also correspond to distinct genotypes.

| 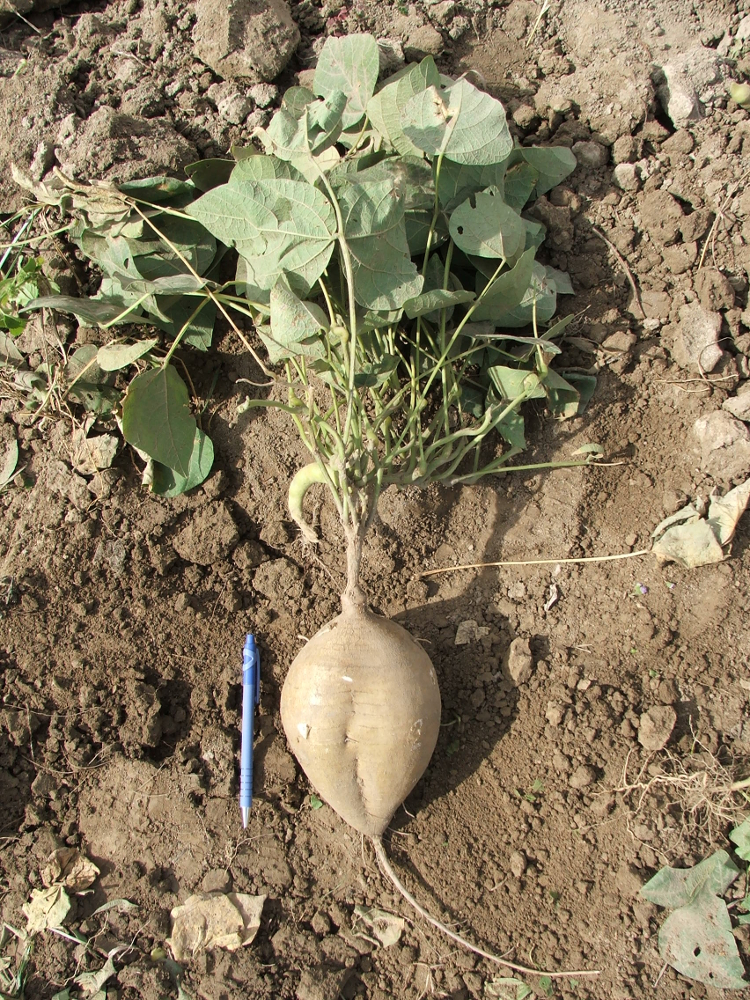 **A** | 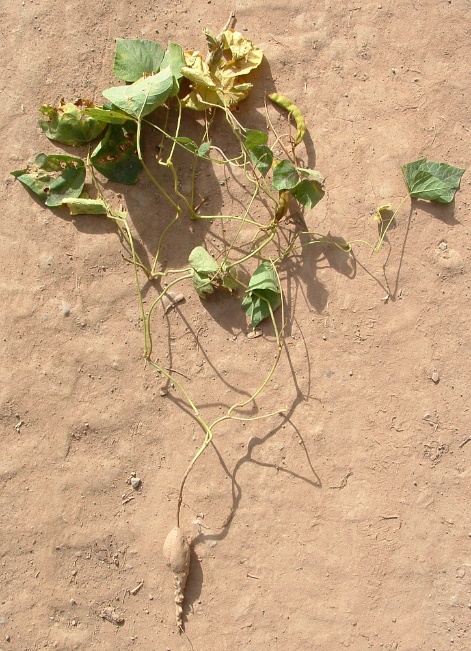  **B** | **C** 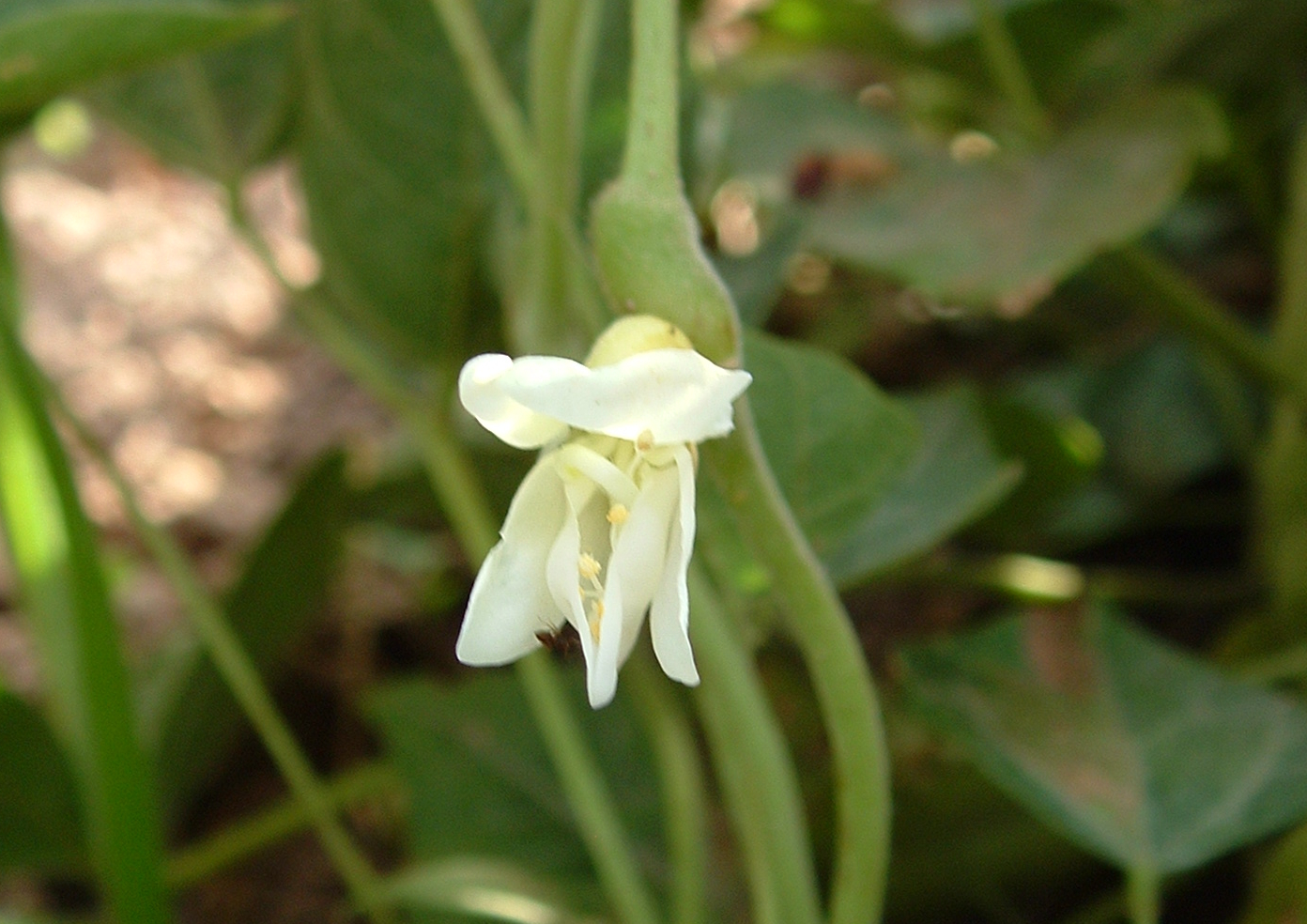 |
| --- | --- | --- |
|  |  | 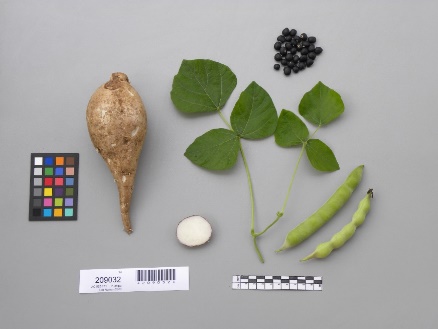  **D** |

**Figure S5.** (A) Bushy Ajipa type from Carapari, southern Bolivia (Photo: B. Soengas, 2010). (B) Twinning type from Anquioma, northern Bolivia (Photo: M. Delêtre, 2010). (C) Ajipa flower (Photo: M. Delêtre, 2010). (D) Ajipa root, leaves, seeds and legume pods (Photo: B. Heider, 2010). Unlike the Ecuadorian Jíquima, Ajipa has entire leaflets.

Subpopulation 14. Yu-L

Subpopulation 15. Yu-H

In Bolivia, *P. tuberosus* is cultivated in the Yungas, a transitional zone between Andean highlands (puna) and Amazon rainforests characterized by warm tropical climate, with no dry season. Tapia and Sørensen (2003) have shown that Bolivian accessions are morphologically distinct from Peruvian Ashipas. Genetic studies further showed that accessions grown at low elevation (Yu-L) differ from those cultivated a high elevation (Yu-H).

1. **Brazil**

Subpopulation 16. Ja

Brazil is a documented area of cultivation for *P. tuberosus*, where it is locally known as Jacatupé or Feijão-macuco. Although its cultivation is now marginal, the crop was commonly grown in during colonial times (Peckolt 1922). It is believed to have been introduced from Peru. All Jacatupé (Ja) accessions were obtained from the Instituto Nacional de Pesquisas Amazônicas (INPA), Manaus, Brazil, but their exact geographic origin is unknown.

Oré Balbín, I., M. Sørensen, L.P. Kvist, O. Delgado Vasquez 2007. Review of the *Pachyrhizus tuberosus* (Lam.) Spreng. cultivar groups in Peru. *Plant Genetic Resources Newsletter*: **151**: 2–13.

Ørting, B., W.J. Grüneberg, and M. Sørensen 1996. Ahipa (*Pachyrhizus ahipa* (Wedd. Parodi) in Bolivia. *Genetic Resources and Crop Evolution* **43**: 435–446.

Peckolt, G. 1922. Jacatupe – *Chácaras and Quintais* **25**: 187–189 and **25**: 275–276.

Sørensen, M. 1988. A taxonomic revision of the genus *Pachyrhizus* (Fabaceae – Phaseoleae). *Nordic Journal of Botany* **8**: 167–192.

Sørensen, M., S. Døygaard, J.E. Estrella, L.P. Kvist, and P.E. Nielsen 1997. Status of the South American tuberous legume *Pachyrhizus* *tuberosus* (Lam.) Spreng. *Biodiversity and Conservation* **6**: 1581–1625.

Tapia, C., and M. Sørensen 2003. Morphological characterization of the genetic variation existing in a Neotropical collection of yam bean, *Pachyrhizus tuberosus* (Lam.) Spreng. *Genetic Resources and Crop Evolution*

[Thiers, B. 2011. Index Herbariorum: a global directory of public herbaria and associated staff. New York Botanical Garden’s Virtual Herbarium. http://sweetgum.nybg.org/ih/. Accessed 5 March 2016](http://sweetgum.nybg.org/ih/)


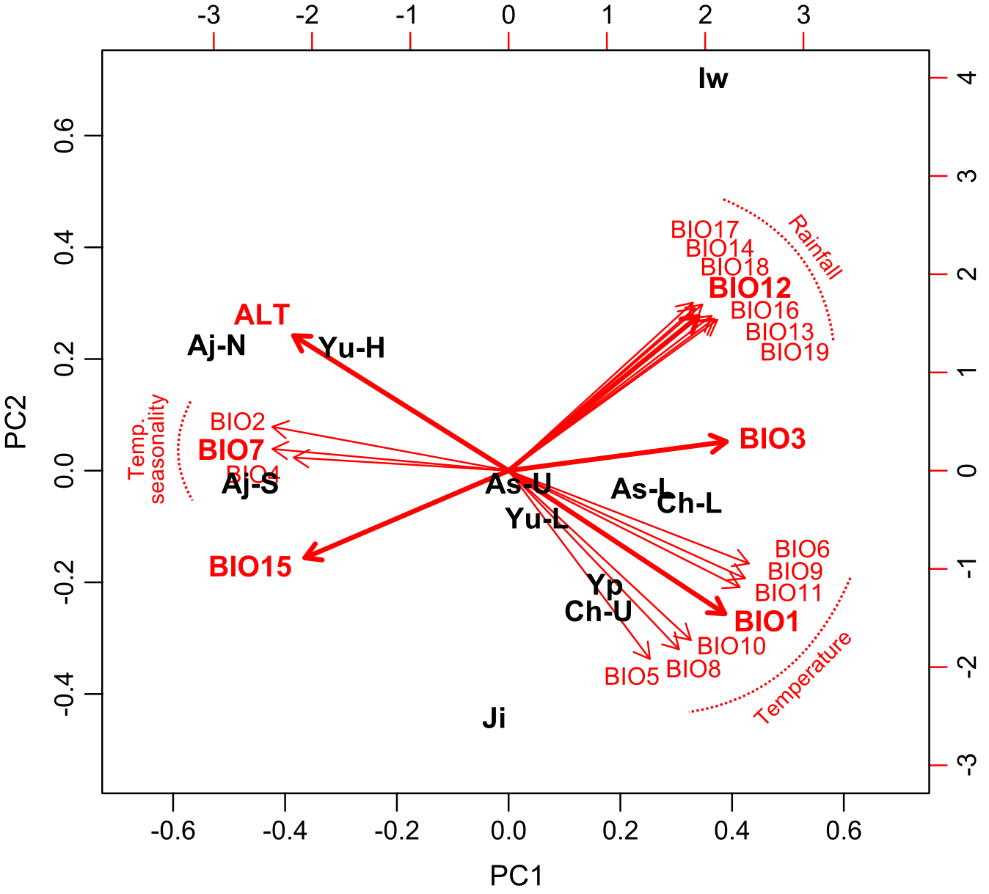


**Fig. S1** Principal Component Analysis of the 19 bioclimatic variables used to identify environmental factors associated with genetic differentiation in *Pachyrhizus* landrace populations. The first two PC axes explained most of the variability (89.3%). PC1 separated landrace populations grown at higher elevations and subject to seasonal variations of temperature (BIO2, BIO4 and BIO7) and rainfall (BIO15), from those cultivated at lower elevations in areas with relatively stable climate. PC2 was mostly drawn by elevation (ALT) and rainfall at one extreme, and temperature and precipitation seasonality (BIO15) at the other. Highly correlated factors related to temperature (BIO1, BIO5, BIO6, BIO8, BIO9, BIO10 and BIO11) and rainfall (BIO3, BIO12, BIO13, BIO14, BIO16, BIO17, BIO18 and BIO19) were subsumed into annual mean temperature (BIO1) and annual precipitation (BIO12), respectively. Similarly, variables related to temperature variability (BIO2, BIO4 and BIO7) were subsumed into temperature annual range (BIO7). Populations are identified as follows: Aj = Ajipa, Ji = Jíquima, Yu = Yungas (Lowlands and Highlands), As = Ashipa, Ch = Chuin, Iw = Iwa. Wild populations were not considered. Key bioclimatic factors retained for landscape genetic analyses are indicated in bold. Bioclimatic variables are coded as follows: BIO1 = Annual Mean Temperature, BIO2 = Mean Diurnal Range (Mean of monthly (max temp - min temp)), BIO3 = Isothermality (BIO2/BIO7) (* 100), BIO4 = Temperature Seasonality (standard deviation *100), BIO5 = Max Temperature of Warmest Month, BIO6 = Min Temperature of Coldest Month, BIO7 = Temperature Annual Range (BIO5-BIO6), BIO8 = Mean Temperature of Wettest Quarter, BIO9 = Mean Temperature of Driest Quarter, BIO10 = Mean Temperature of Warmest Quarter, BIO11 = Mean Temperature of Coldest Quarter, BIO12 = Annual Precipitation, BIO13 = Precipitation of Wettest Month, BIO14 = Precipitation of Driest Month, BIO15 = Precipitation Seasonality (Coefficient of Variation), BIO16 = Precipitation of Wettest Quarter, BIO17 = Precipitation of Driest Quarter, BIO18 = Precipitation of Warmest Quarter, BIO19 = Precipitation of Coldest Quarter.

**Fig. S2** Competing scenarios tested in ABC analyses. Three sets of competing scenarios were compared. In the first set, the Bolivian Ajipa (*P. ahipa*, AC), the Ecuadorian Jíquima (*P. tuberosus*, JI) and Ashipa/Chuin cultivars (*P. tuberosus*, TC) diverged from distinct but closely related wild ancestors (TW) (partial genealogical link; scenarios S1‒S5); in the second set, AC, JI and TC were independently domesticated from the same wild ancestor (weak genealogical link; scenarios S6‒S7); and in the third set, AC and JI diverged from TC (strong genealogical link; scenario S8). The eight competing scenarios were defined based on phylogeographic clusters identified by STRUCTURE and DAPC. Only scenarios compatible with chloroplast DNA were considered. Because herbarium specimens for wild *Pachyrhizus* were scarce, we introduced a fifth ‘ghost’ population to account for unsampled ancestral populations. We assumed that cpDNA haplotype TCA, which was not detected in wild specimens, was probably also present in TW even though it was not sampled. Details on model parameters and prior distributions are given in Table S2.


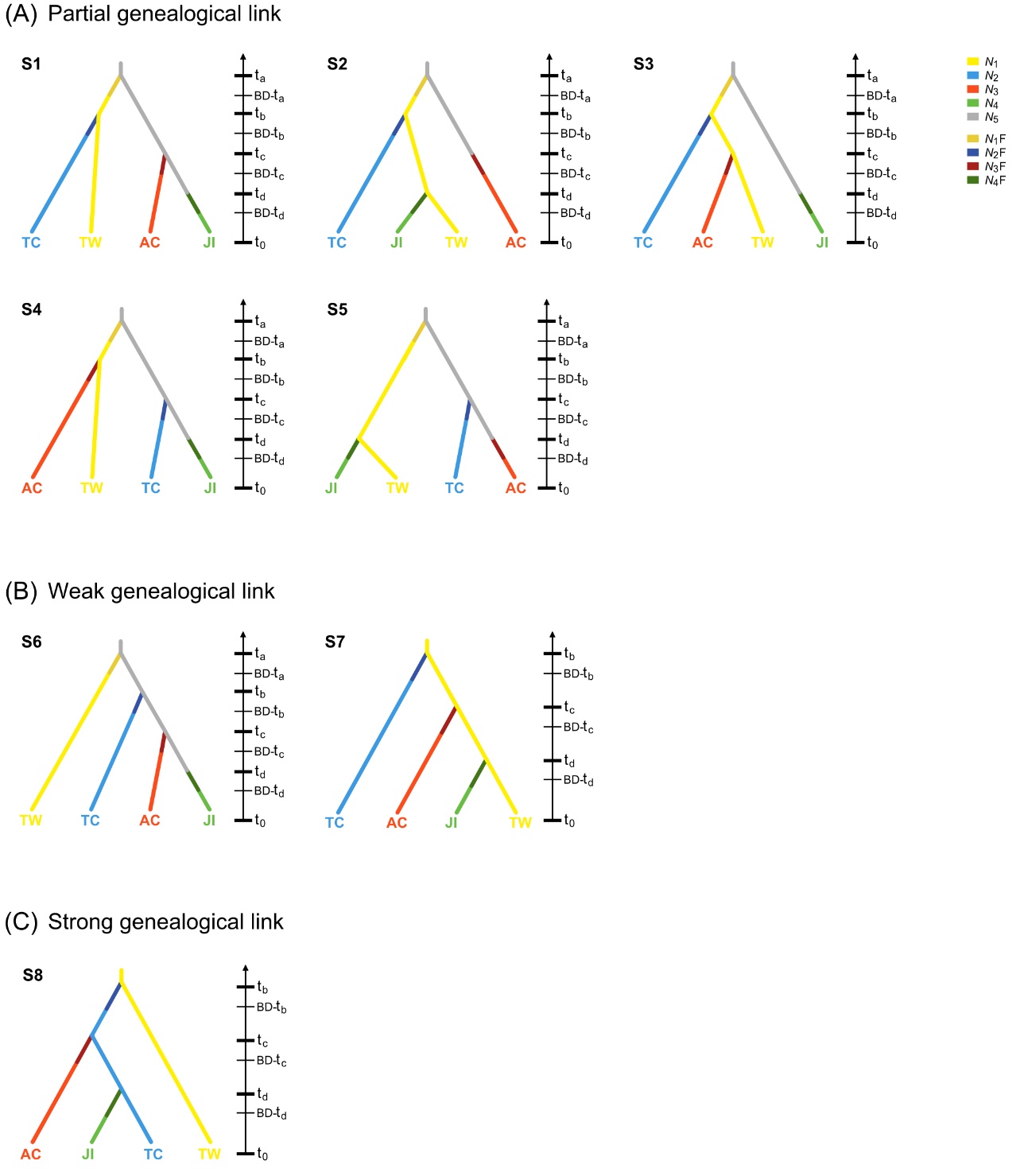


**Table S2** Parameters for prior distribution and conditions used for ABC analyses.

| Parameters | Conditions | Distribution | Interpretation |
| --- | --- | --- | --- |
| *N*_1_ |  | Uniform [10‒20000] | Effective population size TW |
| *N*_2_ |  | Uniform [10‒2000] | Effective population size TC |
| *N*_3_ |  | Uniform [10‒2000] | Effective population size AC |
| *N*_4_ |  | Uniform [10‒2000] | Effective population size JI |
| *N*_5_ |  | Uniform [10‒20000] | Effective population size TW_unsampled_ |
| *t_a_* | *t_a_>t_b_, _ta_>t_c_, _ta_>t_d_* | Uniform [1‒10000] | Divergence time TW_unsampled_→TW |
| *t_b_* | *t_b_>t_c_, _tb_>t_d_* | Uniform [1‒10000] | Divergence time TW→TC |
| *t_c_* |  | Uniform [1‒10000] | Divergence time TC→AC |
| *t_d_* |  | Uniform [1‒10000] | Divergence time TC→JI |
| BD*t_a_* | *t_a_> BDt_a_* | Log-uniform [1‒500] | Bottleneck duration TW_unsampled_→TW |
| BD*t_b_* | *t_b_> BDt_b_* | Log-uniform [1‒500] | Bottleneck duration TW→TC |
| BD*t_c_* | *t_c_> BDt_c_* | Log-uniform [1‒500] | Bottleneck duration TC→AC |
| BD*t_d_* | *t_d_> BDt_d_* | Log-uniform [1‒500] | Bottleneck duration TC→JI |
| *N*_1_F | *N*_1_F< *N*_1_ | Uniform [1‒1000] | Founding population size TW |
| *N*_2_F | *N*_2_F< *N*_2_ | Uniform [1‒1000] | Founding population size TC |
| *N*_3_F | *N*_3_F< *N*_3_ | Uniform [1‒1000] | Founding population size AC |
| *N*_4_F | *N*_4_F< *N*_4_ | Uniform [1‒1000] | Founding population size JI |
| $\bar{\mu}$ |  | Uniform [10^-7^‒10^-4^] | Mutation rate |
| $\bar{p}$ |  | Uniform [0.1‒1.5] | Geometric distribution of the length of mutation events (in number of repeats) |
|  |  |  |  |

As DIYABC does not currently consider insertion/deletion, and as most of DNA variation found in chloroplast DNA sequences consisted of indels, cpDNA showed too little variation to be informative and therefore ABC analyses were carried out with nuclear SSR markers only. Uniform priors were used for all demographic parameters except for bottleneck durations, which were given log-uniform priors. Prior distribution for the time of divergence between populations was set to Un[1–10000], with the upper bound corresponding to the timing of domestication of most Andean crops (~10000 BP). Microsatellite loci were assumed to follow a symmetric generalized stepwise mutation model with a maximum range of 40 contiguous allelic states, a 10^−7^ to 10^−4^ mean mutation rate and a gamma distribution of 2 for individual locus mutation rates to allow for large variance across loci. Single nucleotide insertion/deletion mutations (indels) were not allowed. All parameters were adjusted based on initial runs using the pre-evaluation option of DIYABC.

**Table S3** Pairwise genetic differentiation tests. (A) Weir and Cockerham’s estimator of *F*_ST_ (θ) between *Pachyrhizus* wild and landrace populations (upper-right matrix), and corresponding significance levels (lower-left matrix) after a Benjamini-Hochberg correction for multiple testing (Benjamini and Hochberg 2000). (B) Jost’s estimated genetic differentiation (*D_est_*) between subpopulations.

(A)

|  | AC |  | TC | | | | | | | | |  | TW |
| --- | --- | --- | --- | --- | --- | --- | --- | --- | --- | --- | --- | --- | --- |
|  | Bolivia | | |  | Brazil |  | Ecuador | |  | Peru | |  |  |
|  | Aj |  | Yu |  | Ja |  | Ji | Iw |  | As | Ch |  | (wild) |
| Aj | — |  | 0.751 |  | 0.903 |  | 0.897 | 0.753 |  | 0.653 | 0.841 |  | 0.660 |
| Yu | * |  | — |  | 0.568 |  | 0.734 | 0.319 |  | 0.078 | 0.535 |  | 0.411 |
| Ja | * |  | * |  | — |  | 1.000 | 0.567 |  | 0.497 | 0.877 |  | 0.426 |
| Ji | * |  | * |  | * |  | — | 0.684 |  | 0.539 | 0.858 |  | 0.517 |
| Iw | * |  | * |  | * |  | * | — |  | 0.189 | 0.473 |  | 0.358 |
| As | * |  | *** |  | * |  | * | * |  | — | 0.259 |  | 0.414 |
| Ch | * |  | * |  | * |  | * | * |  | * | — |  | 0.563 |
| Tw | * |  | * |  | * |  | * | * |  | * | * |  | — |

*NS*, not significant, ** P* < 0.05, *** P* < 0.01.

Aj, Ajipa; Yu, Yungas; Ja, Jacatupé; Ji, Jíquima; Iw, Iwa; As, Ashipa; Ch, Chuin; Tw, wild. Yushpe and *P. panamensis*, for which only two accessions were available, were not included.

(B)

|  | AC | |  | TC | | | | | | | | | | | | |  | TW | |
| --- | --- | --- | --- | --- | --- | --- | --- | --- | --- | --- | --- | --- | --- | --- | --- | --- | --- | --- | --- |
|  | Bolivia | | | | |  | Brazil |  | Ecuador | |  | Peru | | | | |  | Ecuador | |
|  | Aj-N | Aj-S |  | Yu-H | Yu-L |  | Ja |  | Ji | Iw |  | As-L | As-U | As-Y | Ch-L | Ch-U |  | Tw-N | Tw-S |
| Aj-N | — | 0.064 |  | 0.381 | 0.228 |  | 0.385 |  | 0.308 | 0.315 |  | 0.263 | 0.239 | 0.311 | 0.235 | 0.280 |  | 0.593 | 0.653 |
| Aj-S | ** | — |  | 0.380 | 0.212 |  | 0.385 |  | 0.308 | 0.312 |  | 0.262 | 0.237 | 0.310 | 0.234 | 0.279 |  | 0.586 | 0.652 |
| Yu-H | ** | ** |  | — | 0.186 |  | 0.226 |  | 0.381 | 0.266 |  | 0.111 | 0.194 | 0.165 | 0.230 | 0.275 |  | 0.614 | 0.708 |
| Yu-L | ** | ** |  | ** | — |  | 0.233 |  | 0.168 | 0.097 |  | 0.048 | 0.022 | 0.141 | 0.020 | 0.065 |  | 0.456 | 0.590 |
| Ja | ** | ** |  | ** | ** |  | — |  | 0.385 | 0.256 |  | 0.221 | 0.237 | 0.234 | 0.234 | 0.279 |  | 0.618 | 0.583 |
| Ji | ** | ** |  | ** | ** |  |  |  | — | 0.247 |  | 0.234 | 0.161 | 0.239 | 0.158 | 0.203 |  | 0.512 | 0.637 |
| Iw | ** | ** |  | ** | ** |  | ** |  |  | — |  | 0.118 | 0.088 | 0.155 | 0.097 | 0.141 |  | 0.391 | 0.533 |
| As-L | ** | ** |  | ** | * |  | ** |  | ** | ** |  | — | 0.081 | 0.060 | 0.101 | 0.146 |  | 0.453 | 0.585 |
| As-U | ** | ** |  | ** | * |  | ** |  | ** | ** |  | ** | — | 0.120 | 0.002 | 0.034 |  | 0.429 | 0.605 |
| As-Y | ** | ** |  | ** | ** |  | ** |  | ** | ** |  | * | ** | — | 0.141 | 0.186 |  | 0.429 | 0.623 |
| Ch-L | ** | ** |  | ** | * |  | ** |  | ** | ** |  | ** | *NS* | ** | — | 0.028 |  | 0.434 | 0.603 |
| Ch-U | ** | ** |  | ** | ** |  | ** |  | ** | ** |  | ** | * | ** | ** | — |  | 0.394 | 0.590 |
| Tw-N | ** | ** |  | ** | ** |  | ** |  | ** | ** |  | ** | ** | ** | ** | ** |  | — | 0.280 |
| Tw-S | ** | ** |  | ** | ** |  | ** |  | ** | ** |  | ** | ** | ** | ** | ** |  | ** | — |

*NS*, not significant, ** P* < 0.05, *** P* < 0.01.

Aj, Ajipa (North and South); Yu, Yungas (Lowlands and Highlands); Ja, Jacatupé; Ji, Jíquima, Iw, Iwa; As, Ashipa (Loreto and Ucayali); Ch, Chuin (Loreto and Ucayali); Tw, wild (North and South). Yushpe and *P. panamensis*, for which only two accessions were available, were not included.

Benjamini, Y., and Y. Hochberg 2000. On the adaptive control of the false discovery rate in multiple testing with independent statistics. *Journal of Educational and Behavioral Statistics* **25**: 60–83.


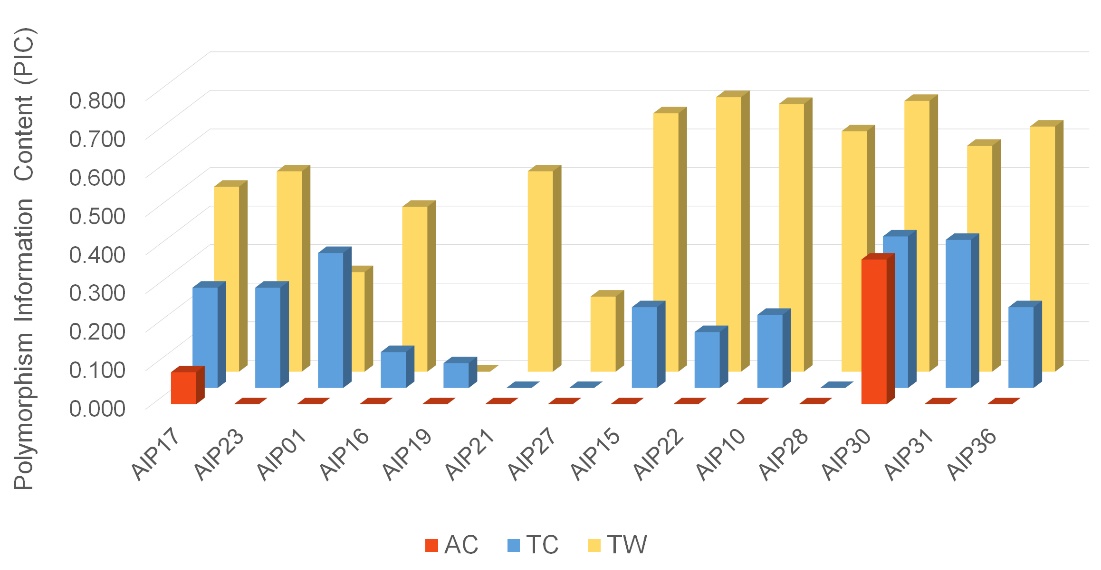


**Fig. S3.** Comparison across species of levels of genetic polymorphism (PIC) at the 14 loci characterized. *P. panamensis*, for which only two samples were available, was not included. Polymorphic information content (PIC) was calculated using Botstein formula (Botstein et al. 1980).

Botstein, D., R.L. White, M. Skolnick, and R.W. Davis 1980. Construction of a genetic linkage map in man using restriction fragment length polymorphisms. *American Journal of Human Genetics* **32**: 314–331.


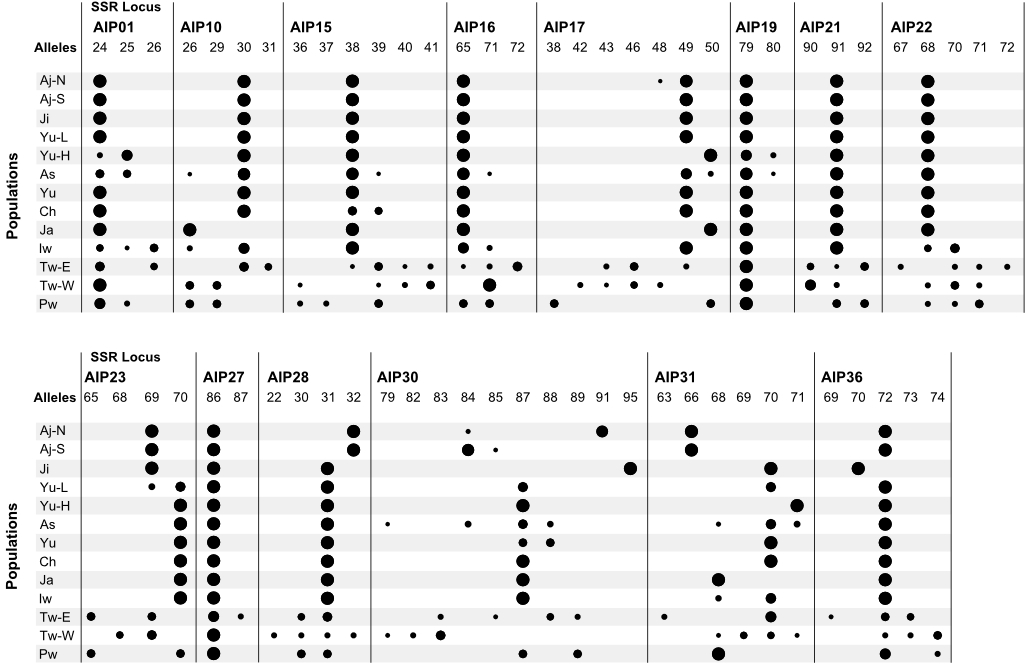


**Fig. S4** Distribution of allelic diversity among South American *Pachyrhizus* populations for the 14 nuclear SSR loci analysed. The presence of an allele is indicated by a dot. The diameter of the dots is proportional to the frequency of the allele in the population. Three loci (AIP05, AIP09, and AIP34) were monomorphic across all species and were excluded from analyses.


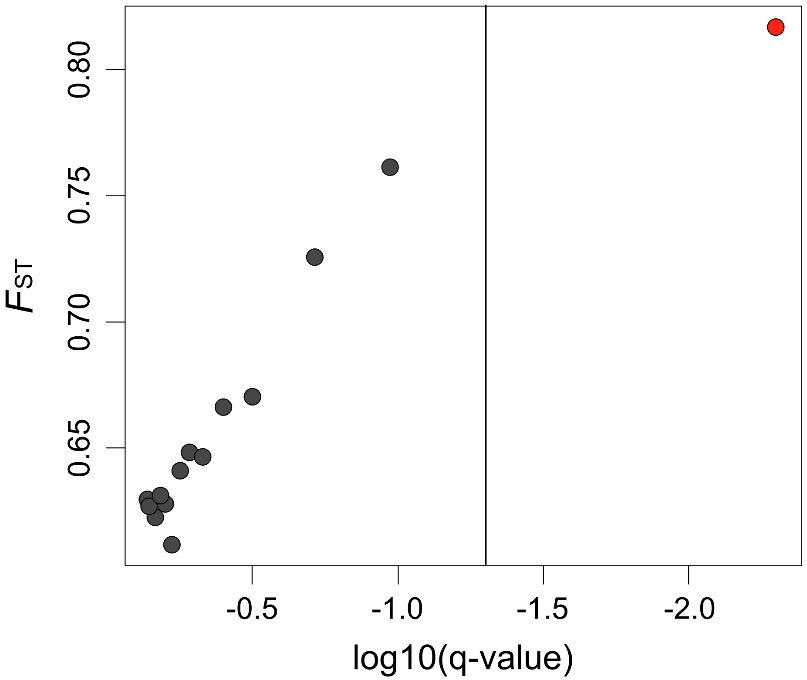


**Fig. S5** Results of the Bayesian outlier detection approach using BAYESCAN. A 1% false discovery rate (FDR) correction (α = 0.01) was applied. The vertical line corresponds to log10(α) = -2. For AIP23 (red dot), log_10_(q value) = -2.3, corresponding to a posterior probability > 0.99 that the locus is under selection (Foll and Gaggiotti 2008). Results were confirmed with a second approach, using the software LOSITAN (Antao et al. 2008).

Foll, M., and O.E. Gaggiotti 2008. A genome scan method to identify selected loci appropriate for both dominant and codominant markers: A Bayesian perspective. *Genetics* **180**: 977–993.

Antao, T., A. Lopes, R.J. Lopes, A. Beja-Pereira, and G. Luikart 2008. LOSITAN: A workbench to detect molecular adaptation based on a *F*_ST_-outlier method. *BMC Bioinformatics*, **9**, 323. DOI: 10.1186/1471-2105-9-323.


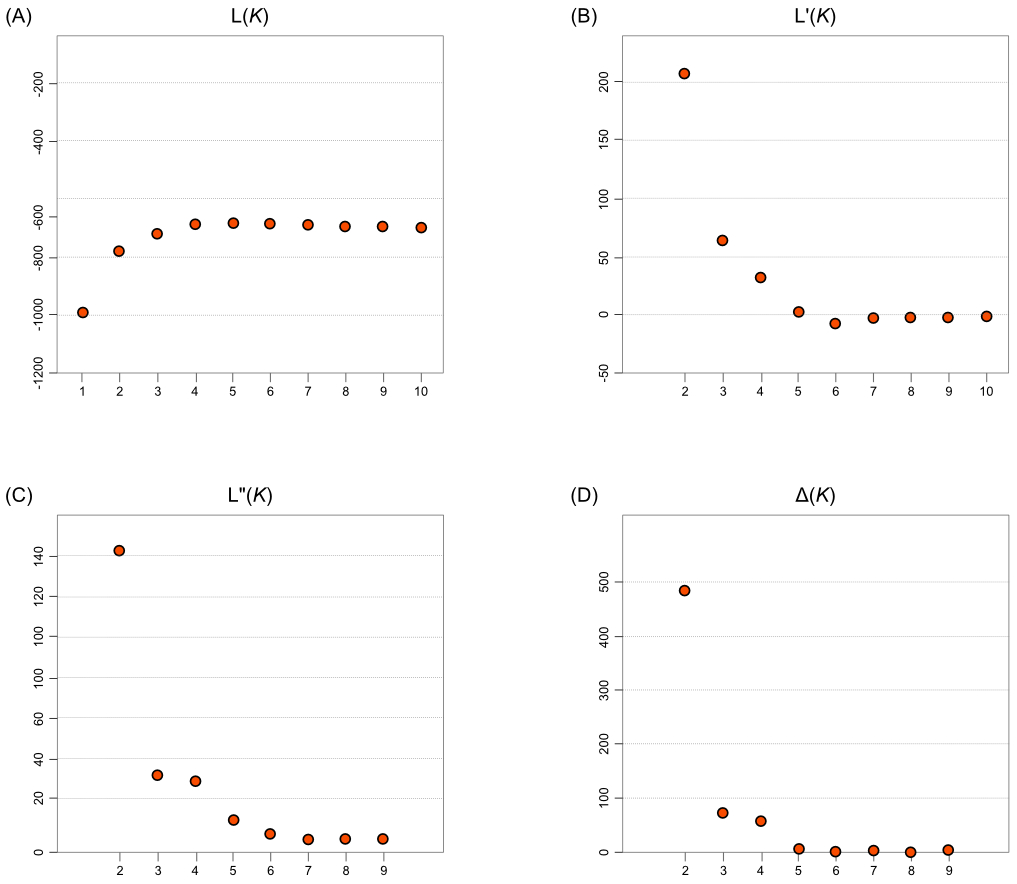


**Fig. S6** Estimation of the optimal number of genetic groups (*K**) detected by STRUCTURE, based on the method of Evanno *et al.* (2005). (A) Mean L(*K*) (± SD) over 10 runs for increasing values of *K*. (B) Mean L’(*K*) (± SD), giving the rate of change of the likelihood distribution. (C) Mean L”(*K*) (± SD), giving the absolute values of the second order rate of change of the likelihood distribution. (D) Δ*K*. Difference between L”(*K*) and L”(*K*+1). Here, the most probable number of populations was *K** = 2. Smaller peaks at *K* = 3 and *K* = 4 indicate the presence of further clustering, which DAPC analyses confirmed.

Evanno, G., S. Regnaut, and J. Goudet 2005. Detecting the number of clusters of individuals using the software STRUCTURE: a simulation study. *Molecular Ecology* **14**: 2611–2620.


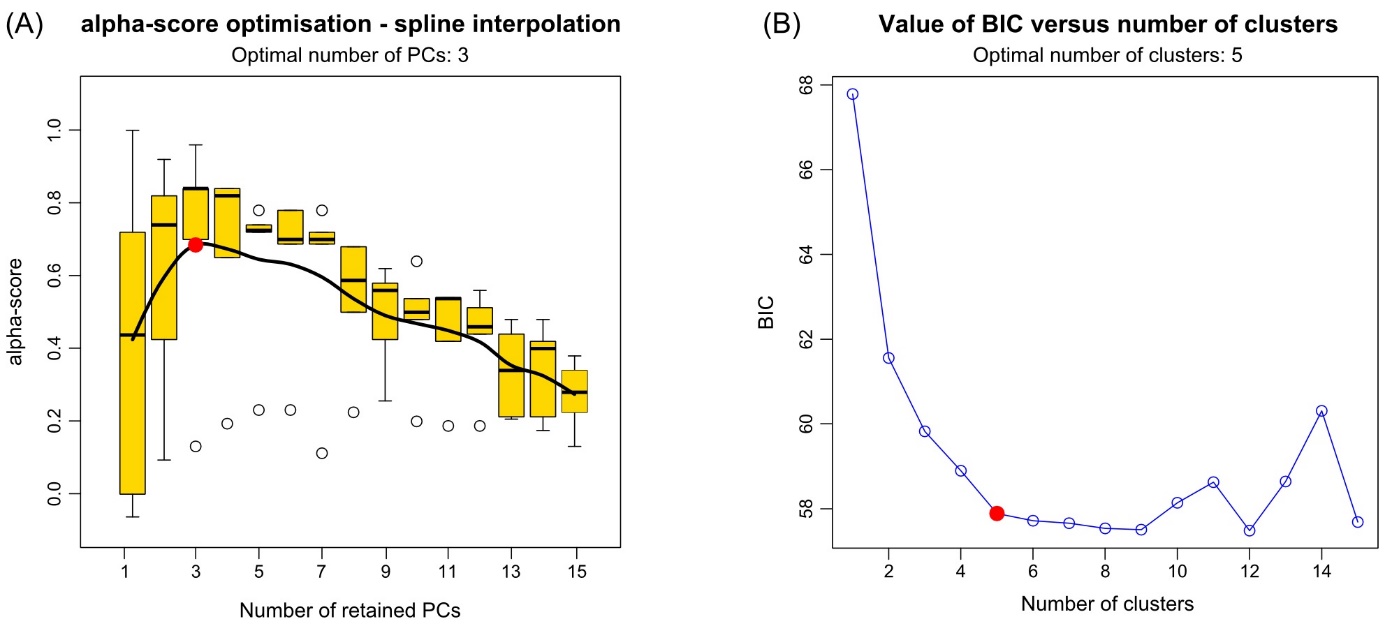


**Fig. S7** DAPC for the whole dataset. (A) Optimization α-score graph. The optimal α-score suggested that seven PCs should be retained for the discriminant analysis. (B) Inference of the number of clusters using the Bayesian Information Criterion (BIC). *K* = 5 (red dot) represented the best summary of the data.


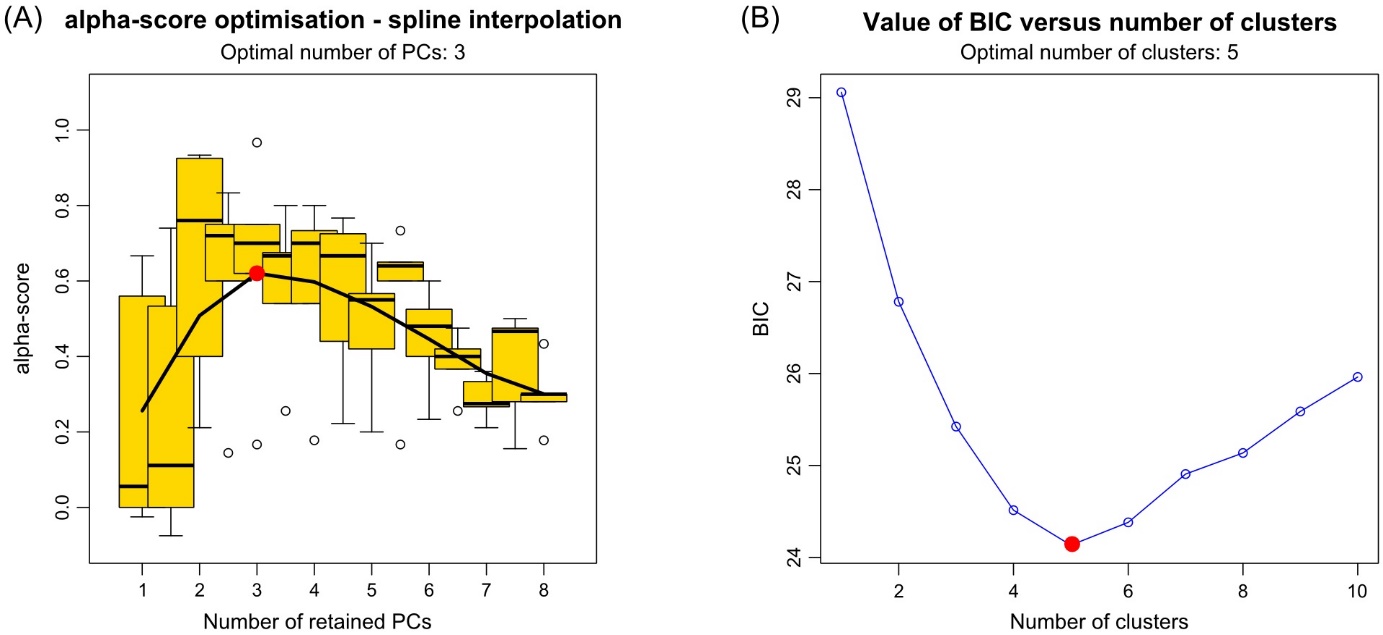


**Fig. S8** DAPC for lineage B. Three PCs were retained for the discriminant analysis. The lowest BIC was obtained for *K* = 5.


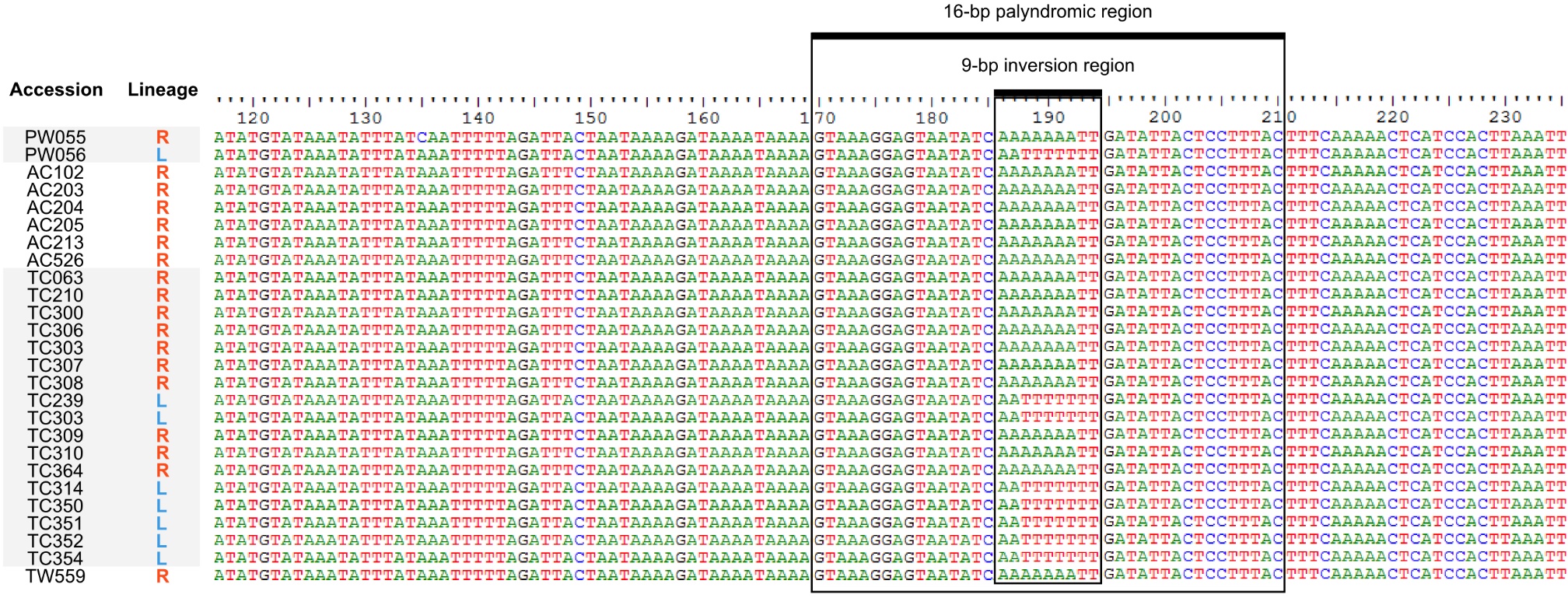


**Fig. S9** Portion of trnH-psbA chloroplast intergenic spacer containing the 9-bp inversion and the 16-bp palindromic flanking sequences.


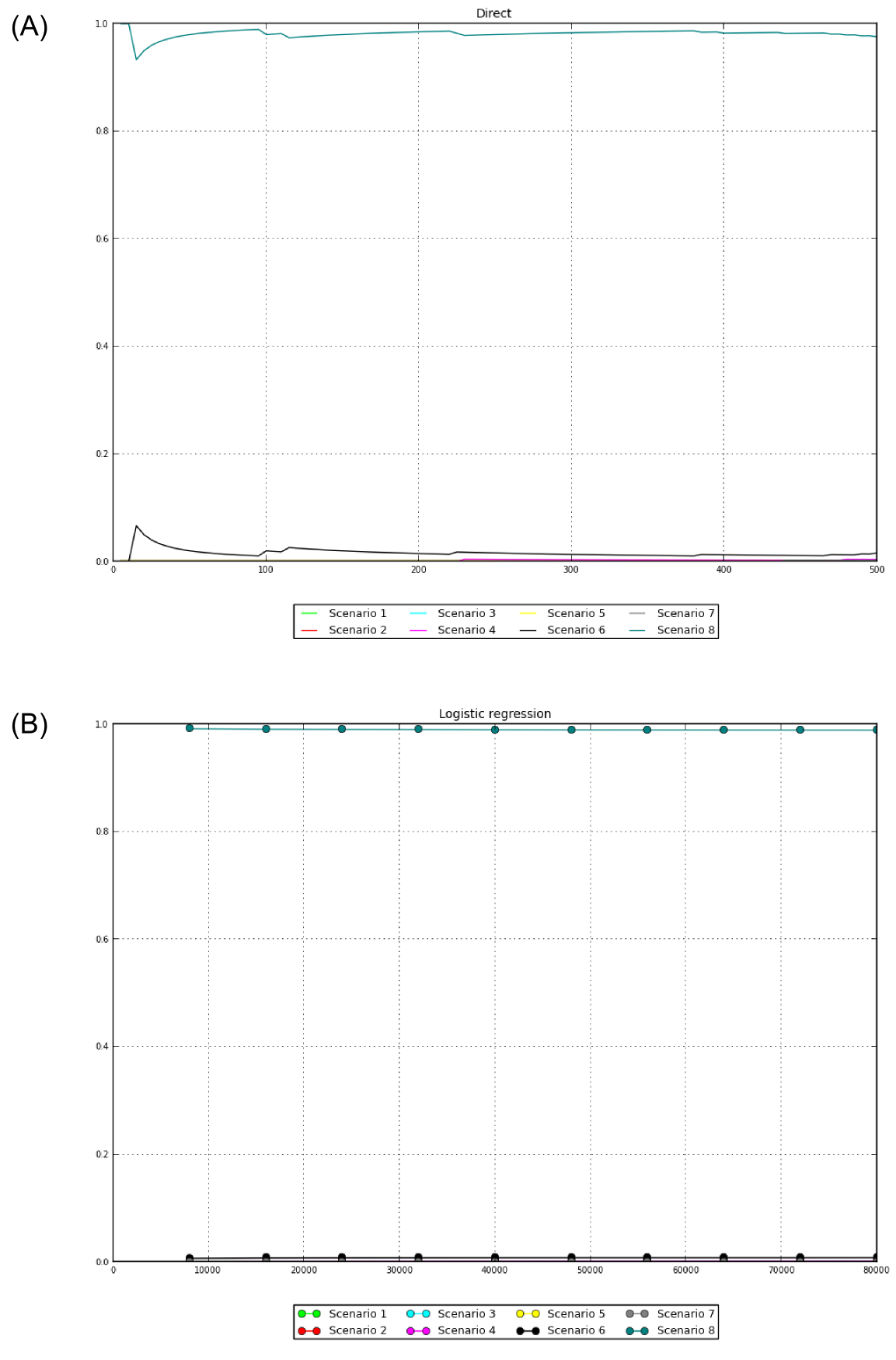


**Figure S10** Comparison of estimated posterior likelihood for the eight competing scenarios using (A) a direct approach based on the 500 closest datasets; (B) a logistic regression approach based on 1% of the closest datasets. Similar results were obtained with AIP23, showing the highest probability for S8 (0.980 [0.972, 0.988]) (data not shown).

**Table S4** Confidence in scenario choice. Type I and type II error rates were estimated from 100 simulated pseudo-observed data sets (PODs) for each of the nine competing scenarios using (A) direct estimate and (B) logistic regression. The best-supported demographic scenario (S8) is shown in bold.

| (A) | "True" scenario | | *Type II error rate* | | | | |  |  |  |
| --- | --- | --- | --- | --- | --- | --- | --- | --- | --- | --- |
|  |  | *Type I error rate* | S1 | S2 | S3 | S4 | S5 | S6 | S7 | **S8** |
|  | S1 | 1 | 49 | 11 | 0 | 6 | 4 | 17 | 12 | **1** |
|  | S2 | 3 | 9 | 54 | 8 | 6 | 4 | 4 | 13 | **2** |
|  | S3 | 0 | 5 | 12 | 41 | 8 | 18 | 3 | 13 | **0** |
|  | S4 | 10 | 5 | 17 | 14 | 45 | 5 | 6 | 4 | **4** |
|  | S5 | 4 | 3 | 8 | 24 | 5 | 41 | 6 | 13 | **0** |
|  | S6 | 4 | 16 | 7 | 9 | 13 | 6 | 24 | 15 | **10** |
|  | S7 | 3 | 3 | 16 | 6 | 6 | 9 | 2 | 57 | **1** |
|  | **S8** | **75** | **1** | **3** | **0** | **10** | **4** | **4** | **3** | **75** |

| (B) | "True" scenario | | *Type II error rate* | | | | |  |  |  |
| --- | --- | --- | --- | --- | --- | --- | --- | --- | --- | --- |
|  |  | *Type I error rate* | S1 | S2 | S3 | S4 | S5 | S6 | S7 | **S8** |
|  | S1 | 0 | 53 | 9 | 4 | 5 | 4 | 18 | 6 | **1** |
|  | S2 | 3 | 8 | 58 | 4 | 8 | 2 | 4 | 11 | **5** |
|  | S3 | 0 | 3 | 13 | 43 | 8 | 17 | 2 | 12 | **2** |
|  | S4 | 8 | 7 | 13 | 14 | 47 | 3 | 9 | 1 | **6** |
|  | S5 | 5 | 4 | 4 | 24 | 3 | 47 | 8 | 10 | **0** |
|  | S6 | 3 | 19 | 9 | 6 | 12 | 8 | 30 | 6 | **10** |
|  | S7 | 1 | 2 | 16 | 8 | 4 | 11 | 4 | 55 | **0** |
|  | **S8** | **80** | **0** | **3** | **0** | **8** | **5** | **3** | **1** | **80** |

**Table S5** Bias and precision of parameter estimates for the best-supported demographic scenario (S8). The performance of parameter estimation was assessed by simulating 100 PODs using medians of demographic parameters drawn from the corresponding posterior distributions.

| Parameter | True value | Mean | MRB | RMSE | RMAE |
| --- | --- | --- | --- | --- | --- |
| *N*_1_ | 15400 | 13950 | -0.068 | 0.310 | -0.533 |
| *N*_2_ | 1522 | 1338 | -0.098 | 0.331 | -0.417 |
| *N*_3_ | 734 | 872 | 0.355 | 1.020 | -2.198 |
| *N*_4_ | 444 | 789 | 1.741 | 3.158 | -6.112 |
| *t_b_* | 7551 | 7715 | 0.074 | 0.370 | -0.411 |
| *t_c_* | 2706 | 3307 | 0.468 | 1.242 | -1.972 |
| *t_d_* | 4174 | 4394 | 0.144 | 0.648 | -0.997 |
| BD*t_b_* | 131 | 84 | 6.453 | 28.841 | -28.346 |
| BD*t_c_* | 85 | 89 | 13.671 | 46.151 | -42.799 |
| BD*t_d_* | 100 | 85 | 13.591 | 47.226 | -46.239 |
| *N*_2_F | 453 | 450 | 0.799 | 2.818 | -2.919 |
| *N*_3_F | 267 | 344 | 4.933 | 24.773 | -29.513 |
| *N*_4_F | 133 | 317 | 10.385 | 45.226 | -65.200 |
| $\bar{\mu}$ | 3.83×10^−5^ | 4.77×10^−5^ | 0.310 | 0.691 | -1.196 |
| $\bar{p}$ | 0.47 | 0.80 | 0.334 | 1.031 | -2.598 |

MRB, mean relative bias; RMSE, square root of the relative mean square error; RMAE, Relative Mean of the Absolute Error (Cornuet et al. 2008).

Cornuet, J.M., F. Santos, M. A. Beaumont, C. P. Robert, J. M. Marin, D. J. Balding, T. Guillemaud et al. 2008. Inferring population history with DIY ABC: a user-friendly approach to approximate Bayesian computation. *Bioinformatics* **24**: 2713–2719.
